# ROS/PI3K/Akt and Wnt/β-catenin signalings activate HIF-1α-induced metabolic reprogramming to impart 5-fluorouracil resistance in colorectal cancer

**DOI:** 10.1186/s13046-021-02229-6

**Published:** 2022-01-08

**Authors:** Shuohui Dong, Shuo Liang, Zhiqiang Cheng, Xiang Zhang, Li Luo, Linchuan Li, Wenjie Zhang, Songhan Li, Qian Xu, Mingwei Zhong, Jiankang Zhu, Guangyong Zhang, Sanyuan Hu

**Affiliations:** 1 Department of General Surgery, Shandong Qianfoshan Hospital, Cheeloo College of Medicine, Shandong University, Jinan, 250014 Shandong Province China; 2 Department of Otolaryngology-Head and Neck Surgery, Shandong Provincial ENT Hospital, Cheeloo College of Medicine, Shandong University, Jinan, Shandong Province China; 3 Department of General Surgery, Qilu Hospital, Cheeloo College of Medicine, Shandong University, Jinan, Shandong Province China; 4 Department of Cardiac Surgery, the First Affiliated Hospital, Sun Yat-sen University, Guangzhou, Guangdong Province China

**Keywords:** HIF-1α, Metabolic reprogramming, Glycolysis, Reactive oxygen species, β-Catenin, Prognostic biomarker, 5-fluorouracil, Chemoresistance, Colorectal

## Abstract

**Background:**

Acquired resistance of 5-fluorouracil (5-FU) remains a clinical challenge in colorectal cancer (CRC), and efforts to develop targeted agents to reduce resistance have not yielded success. Metabolic reprogramming is a key cancer hallmark and confers several tumor phenotypes including chemoresistance. Glucose metabolic reprogramming events of 5-FU resistance in CRC has not been evaluated, and whether abnormal glucose metabolism could impart 5-FU resistance in CRC is also poorly defined.

**Methods:**

Three separate acquired 5-FU resistance CRC cell line models were generated, and glucose metabolism was assessed by measuring glucose and lactate utilization, RNA and protein expressions of glucose metabolism-related enzymes and changes of intermediate metabolites of glucose metabolite pool. The protein levels of hypoxia inducible factor 1α (HIF-1α) in primary tumors and circulating tumor cells of CRC patients were detected by immunohistochemistry and immunofluorescence. Stable *HIF1A* knockdown in cell models was established with a lentiviral system. The influence of both *HIF1A* gene knockdown and pharmacological inhibition on 5-FU resistance in CRC was evaluated in cell models in vivo and in vitro.

**Results:**

The abnormality of glucose metabolism in 5-FU-resistant CRC were described in detail. The enhanced glycolysis and pentose phosphate pathway in CRC were associated with increased HIF-1α expression. HIF-1α-induced glucose metabolic reprogramming imparted 5-FU resistance in CRC. HIF-1α showed enhanced expression in 5-FU-resistant CRC cell lines and clinical specimens, and increased HIF-1α levels were associated with failure of fluorouracil analog-based chemotherapy in CRC patients and poor survival. Upregulation of HIF-1α in 5-FU-resistant CRC occurred through non-oxygen-dependent mechanisms of reactive oxygen species-mediated activation of PI3K/Akt signaling and aberrant activation of β-catenin in the nucleus. Both HIF-1α gene knock-down and pharmacological inhibition restored the sensitivity of CRC to 5-FU.

**Conclusions:**

HIF-1α is a potential biomarker for 5-FU-resistant CRC, and targeting HIF-1a in combination with 5-FU may represent an effective therapeutic strategy in 5-FU-resistant CRC.

**Supplementary Information:**

The online version contains supplementary material available at 10.1186/s13046-021-02229-6.

## Background

Colorectal cancer (CRC) is the third most commonly diagnosed cancer worldwide and ranks second for malignancy-related mortality [1]. Despite recent progress in treatment, the prognosis of CRC remains poor because of the high metastasis and post-intervention recurrence rates [2]. Chemotherapeutic drugs are the main treatment in CRC patients with progression and metastasis, and 5-fluorouracil (5-FU) is a widely used single-agent or key component of systemic chemotherapy for CRC treatment [3, 4]. However, drug resistance can emerge after 5-FU treatment. Although 5-FU pro-drugs and several 5-FU-based combination chemotherapy regimens show beneficial effects, patients with advanced CRC to therapy continue to show poor prognosis due to the development of drug resistance [5]. Therefore, elucidation of the underlying 5-FU resistance mechanisms is critical.

Metabolic reprogramming is one of the important hallmarks of cancer cells [6, 7]. Metabolic reprogramming is characterized by reduced function of the mitochondrial oxidative phosphorylation (OXPHOS) system with energy compensation by glycolysis or the pentose phosphate pathway (PPP) in the presence of abundant oxygen in cancer cells. The primary site of energy production shifts from the mitochondria toward the cytosol [8, 9]. These metabolic transitions ensure a supply of energy and provide building blocks, serving as a key contributor to tumor progression and chemotherapy resistance [10, 11]. Strategies that target metabolic abnormalities will become an effective treatment alternative to enhance drug susceptibility, directly or indirectly [12].

The transcription factor hypoxia inducible factor 1α (HIF-1α) is a key regulator that increases glycolysis and drives tumor development under anoxic conditions [13, 14]. HIF-1α transcriptionally up-regulates glycolytic enzymes and membrane transporters to increase glucose flux and enhance glycolysis [14, 15]. Under normoxia, HIF-1α has also been demonstrated as a major mediator in tumor progression and recurrence [16]. It is worthy to note that the influence of HIF-1α expression on glucose metabolism and resistance of chemotherapy is yet to be fully explored in 5-FU-resistant CRC.

HIF-1α signaling is the classical response to the state of oxygen deficiency [11, 15]. Furthermore, defining the mechanisms of HIF-1α stabilization and activation in normoxia is important to clarify the effect of the complex oxygen environment on the biological behaviors of tumors in vivo. However, the underlying mechanisms are still elusive. Reactive oxygen species (ROS) regulates HIF-1α levels, and HIF-1α also provides a negative feedback to ROS levels [17–19]. The mechanisms of crosstalk between ROS and HIF-1α in regulating glycolysis and 5-FU resistance in CRC have never been reported. Additionally, several studies reported that the Wnt/β-catenin pathway is associated with glycolysis in CRC [20], and up-regulated Wnt/β-catenin promotes resistance to chemotherapy in multiple cancers [21]. HIF-1α is a target of β-catenin, and nuclear β-catenin cooperates with HIF-1α to regulate its transcriptional activity [22]. Based on these facts, it is worth demonstrating whether β-catenin assists HIF-1α to modulate phenotypes in 5-FU resistance in CRC.

Here, we show that the metabolic reprogramming that facilitates 5-FU resistance in CRC arises from HIF-1α upregulation in non-classical ways, by ROS and the Wnt/β-catenin signaling pathway, independently from external oxygen concentrations. Our results provide new insights toward novel therapeutic strategies for suppressing the expression of HIF-1α, which restored the sensitivity of CRC to 5-fluorouracil. Moreover, we provide preliminary data into the potential value of HIF-1α as a biomarker for 5-FU resistance and assessing poor prognosis in CRC patients with 5-FU treatment.

## Materials and methods

### Cell lines

The following colorectal adenocarcinoma cell lines with STR profiling, were obtained from KeyGEN BioTECH, Jiangsu: HCT8 (male), HCT15 (male), HCT116 (male), LoVo (lymph node metastasis, male), SW480 (male), SW1116 (male), HT29 (female), Caco-2 (male), DLD-1 (male), and T84 (lung metastasis, male). The DiFi cell line was a generous gift of Dr. Li Luo. Cells were cultured in Roswell Park Memorial Institute (RPMI)-1640 medium supplemented with 10% fetal bovine serum (FBS), penicillin (100 mg/ml) and streptomycin (100 mg/ml), and cells were subcultured after dissociation with 0.25% trypsin-EDTA once they had reached 80% confluence. In general, all cultures were maintained in a 37 °C 5% CO_2_ incubator, or incubated in a hypoxia chamber at 94% N_2_, 5% CO_2_, and 1% O_2_ for certain experiments.

### Mice

Male athymic BALB/c nude mice (4 weeks old) and male NOD/scid mice (4 weeks old) were purchased from SPF (Beijing) Biotechnology Co., Ltd. and housed in the animal center of Qianfoshan Hospital Affiliated to Shandong University. All procedures were approved by the Institutional Animal Care and Use Committee of Qianfoshan Hospital Affiliated to Shandong University. All animal studies complied with the relevant ethical regulations for animal testing and research.

### Samples from CRC patients and the public GEO database

Fresh and neutral-buffered formalin-fixed tumor samples from 42 patients with CRC were collected from the Department of Gastrointestinal Surgery, Qianfoshan Hospital Affiliated to Shandong University and the Department of Colorectal Surgery, Qilu Hospital of Shandong University (Table S1). At the time of tumor sample collection, 5 mL peripheral venous blood from the cubital vein and 5 mL blood from the tumor reflux veins were also obtained from these patients. An informed consent form was signed by each patient. The Ethics Committee of Qianfoshan Hospital Affiliated to Shandong University granted approval for this study. Datasets involved (GSE104645, GSE69657) were downloaded from the public Gene Expression Omnibus (GEO) database (https://www.ncbi.nlm.nih.gov/gds/).

### Stable acquired 5-FU resistance cell model generation

HCT8, HCT15, HCT116, LoVo, T84, and DiFi cell lines were continuously treated with a gradually increasing concentration of 5-FU rising from 10^− 8^ M to 10^− 4^ M initially. We assessed 5-FU resistance at each dose by calculating the IC_50_ using CCK8 assays. We defined 5-FU resistance as a resistance index (RI, IC_50_ of the WT cells/IC_50_ of the 5-FU-R cells) > 10 after 5-FU treatment of approximately 8 months duration. We succeeded in generating stable cells lines of HCT8 (RI = 396.63), HCT15 (RI = 110.84), and LoVo (RI = 347.60) with acquired 5-FU resistance.

### Knockdown of *HIF1A* and *CTNNB1*

Small interfering RNA (siRNA) duplexes and negative control (NC) targeting *HIF1A* and *CTNNB1* were transfected into cells by overnight incubation using Lipofectamine RNA iMAX reagent according to the manufacturer’s protocol. For lentivirus constructs, the short hairpin RNA (shRNA) for *HIF1A* was cloned into a hU6-MCS-Ubiquitin-EGFP-IRES-puromycin lentiviral (GV248) vector. After lentiviral-mediated transfection and puromycin selection, transfection efficiency was verified by RT-qPCR and WB. The sequences of siRNA and shRNA targeting *HIF1A* or *CTNNB1* are listed in an additional file.

### CCK8 cytotoxicity assay

For cell cytotoxicity assays, 5000 cells per well were seeded into 96-well plates 24 h before treatment. The cells were then treated with the indicated agents for 48-72 h, after which 10% v/v of water-soluble WST-8 dye was added for 0.5-2 h. The absorbance of the formazan was measured at 450 nm using a microplate reader after mixing gently on an orbital shaker for 1 min. All experiments were performed with at least three replicates.

### Cell morphology visualization

Cells were visualized using the CytoPainter phalloidin-iFluor 488 reagent. Fixed and permeabilized cells were stained with phalloidin-iFluor 488 in 1% BSA in PBS for 1 h to label the F-actin. DAPI was used to stain the nuclei. Slides were covered with mounting medium containing DAPI and photographed using an Axio Scope A1 microscope (Zeiss, Germany) at 400× magnification.

### 5-ethynyl-2′-deoxyuridine (EdU) cell proliferation assay

Cells at 5000 per well were plated in 96-well plates for 24 h, and then treated with 10^− 5^ M 5-FU for 48 h. After incubation with 10 μM EdU for a further 2 h, the cells were fixed in 4% paraformaldehyde (PFA) and stained with Click-iT reaction solution at 100 μL per well. Hoechst 33342 was used to stain the nuclei. Images were obtained using an ImageXpress Micro High Content Screening System (Molecular Devices, USA) at 200× magnification, and the ratio of EdU/Hoechst 33342-double-positive cells was quantified.

### Flow cytometry analysis of the cell cycle and apoptosis

Cells were seeded into 6-well plates and treated with or without 10^− 5^ M 5-FU for 48 h, after which they were harvested using trypsin without EDTA. For cell apoptosis analysis, washed cells were immediately stained with PE-conjugated annexin V and 7-AAD and analyzed using a BD FACSAria II instrument (BD Biosciences, USA) and FlowJo software. For cell cycle analysis, cells were fixed in 70% ethanol overnight at − 20 degrees and then resuspended in 1 ml of PBS containing propidium iodide and RNase. Cell cycle stage detection was performed on a FACSAria II machine (BD Biosciences, USA) and data analyzed with ModFit LT software.

### Flow cytometry of Mito-tracker

Cells were cultured in 6-well plates until 80% confluence was achieved. To analyze mitochondria organization, cells were incubated with 1 mL of the working solution of Mito-Tracker for 1 h at 37 °C and kept on ice protected from light. Texas Red fluorescence was visualized using a BD FACSAria II instrument (BD Biosciences, USA) and flow cytometry data were analyzed using FlowJo software.

### Transmission electron microscopy for mitochondrial morphology analysis

Cells were dissociated using trypsin-EDTA solution and collected by centrifugation. They were then fixed in electron microscope fixation liquid for 2 h at room temperature and stored in the dark at 4 °C. Next, fixed samples were sequentially treated with agarose pre-embedding, post-fixation, dehydration, resin penetration, EMBed 821 embedding, polymerization, ultrathin sectioning, and staining. The cuprum grids were observed under an Hitachi HT7800 transmission electron microscope (Hitachi, Japan) and images captured.

### 2-NBDG uptake assay

1 × 10^5^ cells were plated per well in 6-well plates, incubated for 24 h at 37 °C and then treated with the indicated agents for the appropriate time or transfected with *HIF1A* siRNA or control siRNA. Cells were incubated with sugar-free RPMI-1640 medium with 100 μM 2-NBDG for 2 h after starving them in glucose in sugar-free RPMI-1640 medium overnight. After 2-NBDG incubation, washed cells were digested and collected in 1 ml of PBS. The mean fluorescence intensity (MFI) of samples was measured by BD FACSAria II (BD Biosciences, USA) flow cytometry using the FITC channel.

### Lactate release assay

To measure lactate levels secreted into the culture supernatants, 2 × 10^4^ cells per well were seeded into 24-well plates. The medium was refreshed with 1 mL complete RPMI-1640 medium per well for 24 h after treating with the indicated agents. The next day, culture supernatants were harvested and the lactate concentration was measured by colorimetric assays according to the manufacturer’s protocol of the Lactate Assay Kit. Additionally, protein quantitation was measured by the BCA method and lactate release counts were normalized to total protein concentrations.

### ^1^H-nuclear magnetic resonance (^1^H-NMR) platform for intracellular lactate

Cultured cells were washed with ice-cold PBS and removed from the plate by gentle scraping. 2 × 10^7^ cells per sample were collected in triplicate. After removal of the supernatant, 1 mL ice-cold methanol was added and cells were fragmented by ultrasound in an ice bath. Finally, the supernatant was collected after centrifugation, dried and resolubilized in 450 μL D_2_O containing 30 μM 3-(tetramethysilane) propionic acid-2,2,3,3-d4 (TMSP) for spectral referencing. All ^1^H-NMR spectra were recorded at 298 K using a Bruker Avance III 600 MHz spectrometer (Bruker Biospin, Germany), and a standard Bruker noesygppr1d pulse sequence for water suppression was used. The raw ^1^H-NMR spectra were imported into MestReNova 9.0.1 software (Mestrelab Research, Spain) for Fourier transformation, phase and baseline correction.

### Ultra-high pressure liquid chromatography-coupled tandem mass spectrometry (UHPLC-MS/MS) platform for metabolites of central carbon metabolism

Cells (1 × 10^7^ cells per sample) were suspended in 80% methanol, spiked with 20 μL of internal standard solution (2 μg/mL succinic acid-^13^C_4_, 4 μg/mL Fructose-1,6-bisphosphate-^13^C_6_, 10 μg/mL glucose-^13^C_6_), and processed by 5 cycles of ultra-sonication in an ice-water bath. After 30 min at − 20 °C, the cell mixture was centrifuged at 4 °C and 15,000 g for 15 min. The cell-free supernatant was evaporated to dryness and reconstituted in 40 μL of 50% acetonitrile prior to UHPLC-MS/MS analysis. The quality control (QC) sample was obtained by isometrically pooling all the prepared samples. All glycometabolism standards were prepared separately and mixed to a 25 μg/mL standard solution, including glucose, citric acid, cis-aconitic acid, isocitric acid, α-ketoglutaric acid, succinic acid, fumaric acid, malic acid, lactic acid, pyruvic acid, Glucose-6-phosphate (G6P), fructose-6-phosphate (F6P), fructose-1,6-bisphosphate(FBP), ribulose-5-phosphate (Ru5P), xylulose-5-phosphate (Xu5P), sedoheptulose-7-phosphate (S7P), 3-phosphoglyceric acid (3-PGA), glyceraldehyde-3-phosphate (GAP), phosphoenolpyruvic acid (PEP), ribose-5-phosphate (R5P), erythrose-4-phosphate(E4P), and dihydroxyacetone phosphate (DHAP).

The UHPLC-MS/MS analysis was performed on an Agilent 1290 Infinity II UHPLC system coupled to a 6470A Triple Quadrupole mass spectrometer (Santa Clara, USA). Samples were injected into a Waters BEH Amide column (100 mm × 2.1 mm, 1.7 μm) at a flow rate of 0.25 mL/min. The mobile phase consisted of (A) water in 15 mM ammonium acetate at pH 8.5 and (B) 90% acetonitrile. The chromatographic separation was conducted by a gradient elution program as follows: 0-2 min, 90% B; 14 min, 75% B; 15 min, 65% B; 15.2-16.9 min, 50% B; 17-20 min, 90% B. The eluted analytes were ionized by an electro-spray ionization source in positive mode (ESI^−^). The temperatures of the ESI^−^ source drying gas and sheath gas were 300 °C and 350 °C. The flow rates of ESI^−^ source drying gas and sheath gas were 5 and 11 L/minute, respectively. The pressure of the nebulizer was 40 psi, and capillary voltage was 4000 V. Dynamic multiple reaction monitoring (dMRM) was used to acquire data in optimized MRM transition. MassHunter software (version B.08.00, Agilent) was used to control instruments and acquire data.

The raw data were processed on the MassHunter Workstation (version B.08.00, Agilent) using the default parameters with manual inspection asssitance to ensure the qualitative and quantitative accuracies of each compound. The concentrations (C, μg/mL) of metabolites in prepared samples (for determination) were quantified automatically, and finally the output for quantitative calculation of each tube sample in Excel was established with the following formula: *Content* (μg/sample) = C × V, where C is the concentration quantified in the prepared sample (μg/mL), V is the volume of reconstituted solvent (μL).

### Flow sorting on the basis of GLUT1 or MCT4 expression

5 × 10^6^ cells were harvested and washed in pre-cooled PBS. Cells were incubated with anti-GLUT1 (1:50, Abcam) or anti-MCT4 (1:200, Abcam) primary antibodies for 1 h on ice. After washing 3x with cold PBS, cells were incubated with Alexa-conjugated-488 secondary antibody (1:500) for 30 min on ice. At the end of the incubation, cells were sorted on a BD FACSAria III instrument (BD Biosciences, USA) for high and low expression of GLUT1 or MCT4. Sorted cells were then cultured and CCK8 cytotoxicity assays performed.

### Measurement of oxygen consumption rates (OCR) and extracellular acidification rates (ECAR)

1 × 10^4^ cells were seeded into XFe96 cell culture plates in complete RPMI-1640 medium and incubated at 37 °C overnight in 5% CO_2_. To equilibrate temperature and pH of the detection system, cells were washed with assay RPMI-1640 medium and incubated at 37 °C for 1 h in a CO_2_-free incubator before assessment. OCR and ECAR were detected with an Agilent Seahorse XFe96 extracellular flux analyzer (Agilent Technologies, USA). To examine mitochondrial respiratory activity, cells were treated with oligomycin (1.5 μM), FCCP (1 μM) and rotenone/antimycin A (0.5 μM) using a Seahorse XF Cell Mito Stress Test Kit. For assessment of glycolytic activity, cells were treated with glucose (100 mM), oligomycin (10 μM) and 2-deoxy-D-glucose (2-DG, 500 mM) using a Seahorse XF Glycolytic Rate Assay Kit in sequence. All experiments were done in seven replicas each time and data expressed as means with SEM.

### Fluorometric measurement of ATP production

4 × 10^5^ cells per well were seeded into 6-well plates until 70-90% confluence was achieved. Cells were lysed and cell lysates used for detecting ATP and total protein concentrations. ATP concentrations were measured using a luminometer and determined based on the standard curve. The results were normalized to protein content (BCA assay).

### Flow cytometry for intracellular ROS levels

When cells in T25 flasks had reached 60% confluence, they were probed using 10 μM 2′,7′-dichlorodihydrofluorescein diacetate (DCFH-DA) incubated at 37 °C for 20 min. Fluorescence signals in the FITC channel were acquired on a BD FACSAria II (BD Biosciences, USA) instrument for measurement of intracellular ROS. Data were analyzed using FlowJo software.

### Colorimetric measurement of enzyme activities

Lactate dehydrogenase (LDH), catalase (CAT), glutathione peroxidase (GPx), and superoxide dismutase (SOD) activities were measured by colorimetry, following the manufacturer’s protocols. Enzyme activities were normalized to total protein levels (BCA assay).

### Protein extraction and Western blotting

To extract total proteins, cells were lysed in RIPA buffer containing a protease inhibitor and a phosphatase inhibitor. In addition, nuclear, cytosolic, and membrane proteins were extracted using a Nuclear Protein Extraction Kit or a Membrane Protein Extraction Kit according to the manufacturer’s instructions. Protein solutions were boiled in 5 × loading buffer at 98 °C for 10 min and then resolved by SDS-PAGE. All the primary antibodies used in the experiment were: anti-CDK2 (1:1000, Proteintech), anti-Cyclin D1 (1:1000, Proteintech), anti-p21 (1:2000, Proteintech), anti-Bcl-XL (1:1000, Proteintech), anti-Cleaved caspase 3 (1:500, Affinity), anti-Cleaved caspase 9 (1:1000, Affinity), anti-NDUFB8 (1:2000, Abcam), anti-SDHB (1:100000, Abcam), anti-UQCRC1 (1:1000, Proteintech), anti-COX4 (1:2000, Abcam), anti-ATP5F1 (1:500, Proteintech), anti-GLUT1 (1:5000, Abcam), anti-GLUT2 (1:5000, Abcam), anti-GLUT3 (1:1000, Abcam), anti-GLUT4 (1:500, Abcam), anti-MCT1 (1:1000, Abcam), anti-MCT2 (1:500, Proteintech), anti-MCT4 (1:200, Santa), anti-LDHA (1:1000, Proteintech), anti-HK2 (1:1000, Abcam), anti-GAPDH (1:10000, Proteintech), anti-ENO1 (1:1000, Abcam), anti-PKM2 (1:500, CST), anti-G6PD (1:1000, Abcam), anti-HIF-1α (1:500, Abcam), anti-Catalase (1:2000, Proteintech), anti-GPx1 (1:2000, Abcam), anti-SOD1 (1:50000, Abcam), anti-SOD2 (1:1000, Abcam), anti-PI3K (1:1000, Affinity), anti-Phospho-PI3K (1:1000, Affinity), anti-AKT (1:500, Abcam), anti-Phospho-AKT (1:1000, Abcam), anti-β-catenin (1:5000, Proteintech), anti-Axin2 (1:1000, Abcam), anti-Dvl1 (1:1000, Proteintech), anti-TCF1 (1:1000, Proteintech), anti-TCF4 (1:1000, Proteintech), anti-β-Actin (1:5000, Proteintech), anti-Lamin B1 (1:2000, Proteintech). Detailed antibody information was listed in additional file. Primary antibodies were detected by goat anti-rabbit/mouse IgG (H + L), horse radish peroxidase (HRP)-conjugated secondary antibodies, and reacted with a chemiluminescent HRP substrate. Chemiluminescence images were captured using an Amersham Imager 680 (GE Healthcare, USA).

### Co-immunoprecipitation (co-IP) analysis

Pierce Co-IP Kits (Thermo Scientific) were used for the isolation of natural protein complexes. Our approach is briefly summarized in the following steps. Primary antibodies against β-catenin (0.5 μg/μL, Proteintech) or HIF-1α (0.165 μg/μL, Abcam) were first covalently immobilized to AminoLink Plus coupling resins for 2 h at room temperature in a tube rotator, with IgG serving as the negative control. Cells were solubilized in lysis buffer (Tris 0.025 M, NaCl 0.15 M, EDTA 0.001 M, NP-40 1%, and glycerin 5%), and 500 μL of the cell lysates incubated with pre-prepared anti-β-catenin or anti-HIF-1α primary antibody-conjugated resins overnight at 4 °C in a tube rotator. After incubation, the natural protein complexes were eluted and analyzed by Western blotting with anti-β-catenin (1:5000, Proteintech), anti-HIF-1α (1:500, Abcam), anti-TCF1 (1:1000, Proteintech), and anti-TCF4 (1:1000, Proteintech) primary antibodies as described previously.

### Total RNA extraction and real-time quantitative PCR (RT-qPCR)

Total RNA was extracted with TRIzol reagent (TaKaRa) according to the manufacturer’s instructions. Extracted total RNA was measured on a NanoDrop spectrophotometer (NanoDrop Technologies, USA) and reverse transcribed to cDNA using ReverTra Ace qPCR RT Kit (TOYOBO). The cDNA was detected using a mixed system containing 1 μL cDNA, 0.8 μL gene-specific primers, and 5 μL SYBR Green Realtime PCR Master Mix (TOYOBO) using a LightCycler 480 II instrument (Roche, Switzerland). Relative mRNA quantification was performed by the ΔΔCt method and the housekeeping gene *ACTB* was used as an internal reference. Detailed information of primer sequences is listed in the additional files.

### Immunocytofluorescence

1 × 10^5^ cells were seeded on cell climbing slices coated with poly-D-lysine in 24 well plates. Prepared cells were fixed in 4% PFA for 20 min, permeabilized with 0.5% Triton X-100 for 5 min, and blocked in 10% goat serum for 1 h at room temperature. The following primary antibodies were incubated overnight at 4 °C: anti-LDHA (1:250, Proteintech), anti-β-catenin (1:200, Proteintech), or anti-HIF-1α (1:200, Abcam; 1:100 Proteintech). Next day, cells were incubated with secondary antibodies for 1.5 h at room temperature: CoraLite594-conjugated goat anti-rabbit IgG(H + L) (1:250, Proteintech), CoraLite488-conjugated goat anti-mouse IgG(H + L) (1:250, Proteintech). Cell climbing slices were mounted on slides with mounting medium containing DAPI and photographed by a TCS SP8 confocal laser scanning microscopy (Leica, Italy) or an Axio Scope A1 microscope (Zeiss, Germany) at 400× magnification.

### Subcutaneous cell-derived xenograft (CDX) nude mouse model

Male athymic BALB/c nude mice were raised under 12 h light/12 h dark cycles in an SPF environment. We subcutaneously implanted 5 × 10^6^ WT or 5-FU-R cells with or without stable knockdown of sh*HIF1A* into the right forelimb underarm of 4 week-old nude mice. One week after subcutaneous injection, the mice were intraperitoneally injected with 5-FU (25 mg/kg, three times a week), or saline as a control. In the experiments on HIF-1α pharmacological inhibition, one week after subcutaneous injection, both WT or 5-FU-R cell-bearing nude mice were then randomly divided into 4 groups: control, 5-FU, IDF-11774, and 5-FU + IDF-11774. 5-FU was intraperitoneally injected at the dose of 25 mg/kg three times a week, IDF-11774 was intraperitoneally injected at the dose of 30 mg/kg twice a week, and saline as a control. Subcutaneous tumor-bearing mice were continuously monitored, and tumor volumes (*V* = *L* × *W*^2^/2, where *L* is the length and *W* is the width) were assessed. Mice were sacrificed under anesthesia when the tumor reached a diameter of about 1.5 cm.

### Patient derived xenograft (PDX) nude mouse model

Tumor tissues derived from a rectal cancer patient (male, Dukes’ C) received neoadjuvant chemotherapy containing 5-FU and subsequent surgery. Fresh well-trimmed tumor tissues were cut into pieces about 3 mm in size and immediately placed in RPMI-1640 medium supplemented with 50% FBS and penicillin/streptomycin. We subcutaneously implanted tumor pieces into the right forelimb underarm of 4 week-old NOD/scid mice, and xenografts were harvested when they reached a diameter of about 1.5 cm. They were then cut up and implanted again into fresh NOD/scid mice. When these xenografts had been growing for about 20 days, mice were divided into four groups: control (saline), 5-FU (25 mg/kg, three times per week), IDF-11774 (30 mg/kg, twice per week), and 5-FU with IDF-11774. Mice were sacrificed under anesthesia when the PDXs reached a diameter of about 1.5 cm.

### Immunohistochemistry (IHC)

CDX/PDX tumors and CRC tissues of patients were fixed in 4% PFA, embedded in paraffin, and sectioned. Tumor sections were dewaxed with dewaxing agent and dehydrated in graded alcohol concentrations. After antigen retrieval with heated citrate buffer and blocking with 10% goat serum, the following primary antibodies were incubated overnight at 4 °C: anti-Ki-67 (Proteintech, 1:5000), anti-HIF-1α (1:200, Abcam), anti-GLUT1 (1:500, Abcam), anti-HK2 (1:100, Abcam), anti-PKM2 (1:200, CST), anti-LDHA (1:200, Proteintech), anti-MCT4 (1:50, Santa), anti-Phospho-AKT (1:100, Abcam), anti-β-catenin (1:1000, Proteintech), and anti-TCF1 (1:200, Proteintech). Next day, the sections were incubated with HRP-conjugated anti-rabbit/mouse IgG and detected by 3,3′-diaminobenzidine (DAB) staining. The nuclei were stained with hematoxylin. The stained sections were photographed using an Axio Scope A1 microscope (Zeiss, Germany) at 200× magnification.

### In vivo glucose uptake assay

WT or 5-FU-R cell-bearing nude mice were injected with 10 nmol IRDye 800CW 2-DG Optical Probe (Li-Cor Biosciences) via the tail vein, and then imaged using an IVIS Kinetic (Caliper Life Sciences, USA) small animal imaging system with a cooled Hamamatsu ORCA-R2 camera (Hamamatsu, Japan) 24 h later. Probe signals were displayed as pseudo-colored bioluminescent images and merged with grey-scale white light images of the mice. Circular ROIs were drawn over the areas and quantified, and the results are reported as total radiant efficiency.

### Lactate-magnetic resonance spectroscopy (MRS)

Tumor lactate concentrations of WT or 5-FU-R cell-bearing nude mice were quantified with MRS. The data were acquired on a Siemens Skyra 3.0 T MRI scanner (Siemens Skyra, Germany) using an 8-channel small animal special coil (mouse coil) for reception of the signal. T2-weighted anatomical images were acquired using a standard 3D sequence with an isotropic voxel size of 1 mm. Subsequently, the lactate spectrum was acquired from a single voxel snug-fit to the tumor with TR/TE of 1700 ms/135 ms. The acquisition duration was set to 853 ms with a bandwidth of 1200 Hz.

### Enrichment and detection of circulating tumor cells (CTCs)

Tumor-reflux venous blood was collected from patients with CRC during surgery, and peripheral venous blood was collected at the same time. Three mL normal saline (0.9%) and 200 μL PFA (8%) were added to 5 mL EDTA-anticoagulated whole blood samples, which were then transferred to a filter with an 8 μm diameter aperture membrane. CTCs were captured on the membrane by a CTCBIOPSY device (YZYBIO Company, China). Given that captured cells on the membrane included CTCs but also normal blood cells, candidate CTCs were distinguished by Wright’s staining. After destaining, detection of HIF-1α expression was performed by immunofluorescence analysis.

### Statistical analysis

All data are shown as means ± standard errors of the mean (SEM). A two-tailed Student’s t-test was used to compare variables of two groups, and one-way or two-way ANOVA were performed for multi-group comparisons. Correlation analysis used Pearson or Spearman correlation analysis. Patient survival data related to HIF-1α expression was evaluated by the Kaplan-Meier method with survival analysis using Log-rank (Mantel-Cox) testing. Significance of differences is marked ns = not significant, * *p* < 0.05, ** *p* < 0.01, and *** *p* < 0.001, and all *p* values < 0.05 were considered statistically significant. Statistical details are included in the respective figure legends.

## Results

### Establishment and characterization of acquired 5-FU-resistant CRC cell lines

**Fig. 1:**
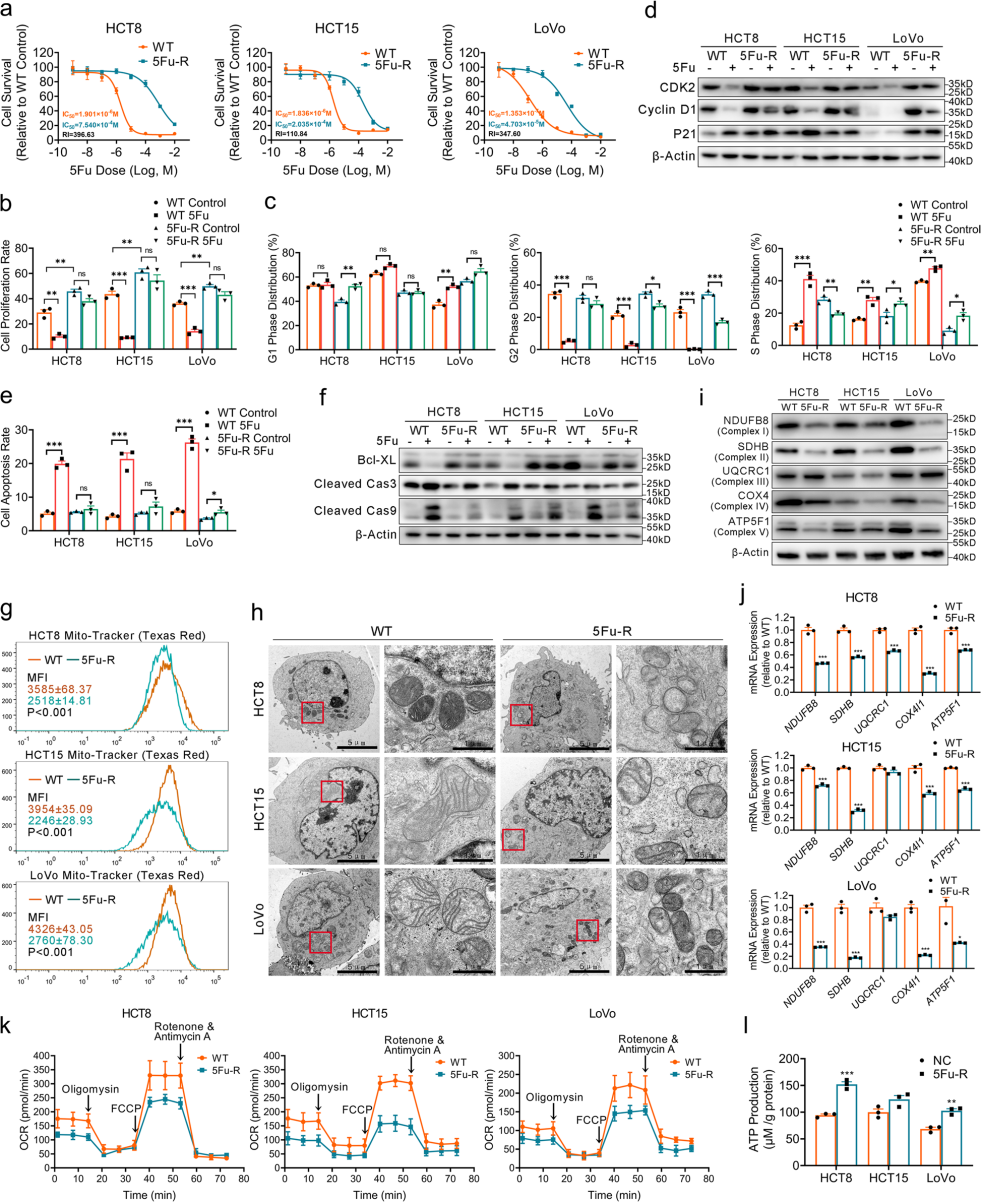
Establishment and characterization of the acquired 5-FU-R CRC cell line models. **a** CCK8 assays to assess 5-FU sensitivity of 5-FU-R CRC cells compared with WT CRC cells, and cells cultured with increasing concentrations of 5-FU for 72 h. All experiments were performed with 6 replicates. **b** Proportion of EdU-positive cells with or without 10^− 5^ M 5-FU treatment. **c** Cell cycle data for G1 phase, G2 phase and M phase distribution with or without 10^− 5^ M 5-FU treatment. **d** Western blots of cell cycle markers. β-Actin was used as the internal reference. **e** Apoptosis with or without 10^− 5^ M 5-FU treatment. Both early and late apoptotic cells are shown. **f** Western blots of apoptosis markers. β-Actin was used as the internal reference. **g** Mitochondria marked by Mito-tracker (Texas Red). Data are mean MFI (Texas Red channel) ± SEM. **h** Typical images of the ultrastructure of mitochondria by transmission electron microscopy (TEM). Scale bar = 5 μm/1 μm. **i** Western blots of five enzymes of the mitochondrial respiratory chain. β-Actin was used as the internal reference. **j** RT-qPCR analysis of mitochondrial enzyme genes in 5-FU-R CRC cells relative to WT CRC cells. *ACTB* was used as the internal reference. **k** 5-FU-R and WT cells were distributed into in 96-well Seahorse assay plates and oligomycin (1.5 μM), FCCP (1 μM) and rotenone/antimycin A (0.5 μM) were successively added to measure OCR. **l** ATP production of 5-FU-R CRC cells relative to WT CRC cells detected by fluorometric analysis. ATP production was normalized to total protein concentration. For all studies n was ≥3. Data are means ± SEM. Bar chart data were compared by Student’s t-test or ANOVA (ns = not significant, * *p <* 0.05, ** *p <* 0.01, and *** *p <* 0.001)

To investigate the influence of metabolic reprogramming on 5-FU resistance in CRC, we generated three acquired 5-FU resistance (5-FU-R) CRC cell line models (HCT8, HCT15, and LoVo). We cultured wild-type (WT) cells with increasing 5-FU concentrations (10^− 8^ M to 10^− 4^ M) over approximately 8 months (Fig. S1a). We calculated the half maximal inhibitory concentration (IC_50_) and found that 5-FU-R cells had resistance indexes of 100 to 500 at the end of 5-FU induction and measured after 2 weeks of drug withdrawal (Fig. 1a). WT cells usually grow and fuse into pieces, and the intercellular space was less distinct in in vitro culture. Phalloidin staining showed the 5-FU-R cells were more independent and stretched out compared with WT cells (Fig. S1b). EdU assay revealed an increase in 5-FU-R cell proliferation under control and 5-FU treatments compared with control cells with the respective treatments; treatment with 5-FU had no impact on 5-FU-R cells compared with untreated 5-FU-R cells (Fig. 1b and Fig. S1c). We further found that 5-FU treatment induced cell cycle arrest of WT cells in S phase while it had no effect on 5-FU-R cells (Fig. 1c and Fig. S1d). Western blot analysis showed WT and 5-FU-R cells had different regulations on cell cycle governors under control and 5-FU treatment, like CDK2, cyclin D1 and p21 (Fig. 1d). Moreover, WT cells displayed a higher apoptosis rate 5-FU treatment compared with 5-FU-R cells (Fig. 1e and Fig. S1e). Similar results were observed with the apoptosis markers Bcl-XL, cleaved caspase 3 and cleaved caspase 9 (Fig. 1f). Together, these findings indicate that 5-FU-R CRC cells show low response to high-dose 5-FU treatment and confirm successful in vitro cell model establishment.

### Mitochondrial damage drives the metabolic shift from OXPHOS to alternative pathways

Mitochondria are the center of cellular energy metabolism [23]. To determine whether 5-FU-R CRC cells showed altered glucose metabolism, we evaluated the status of mitochondria. Mitochondria were labeled using Mito-tracker to determine mitochondrial content. Flow cytometry revealed that 5-FU-R cells displayed markedly decreased mean fluorescence intensities compared with WT cells (Fig. 1g). We next visualized the mitochondria at the ultrastructural level by transmission electron microscopy. The mitochondria of 5-FU-R cells were pyknotic and showed vacuolization, ruptured membrane, cristae disorganization, fusion and low numbers compared with WT cells (Fig. 1h). The abnormal mitochondrial number and distribution in 5-FU-R cells pointed to potential mitochondrial dysfunction. The top biological function of mitochondria is oxidative metabolism, and five enzyme complexes of the mitochondrial respiratory chain are responsible for generating ATP by OXPHOS. The gene and protein expressions of mitochondrial enzymes (NDUFB8, SDHB, UQCRC1, COX4, and ATP5F1) were significantly down-regulated in 5-FU-R cells compared with controls (Fig. 1i, j). To directly assess mitochondrial respiration, we measured OCR. The 5-FU-R cells demonstrated significantly decreased OCR compared with WT cells (Fig. 1k). Basal respiration, maximal respiration, and spare capacity were also significantly reduced in 5-FU-R cells (Fig. S1f). These results show that 5-FU resistance is accompanied by abnormal mitochondrial structure and function. We observed that 5-FU-R cells did not exhibit reduced ATP production compared with WT cells (Fig. 1l). This suggests that 5-FU-R cells probably develop a metabolic shift and activate alternative pathways to increase ATP production to compensate for mitochondrial damage.

### Increased glucose and lactate metabolism fuel 5-FU resistance in 5-FU-R CRC cells

Energy metabolism plays an important role in the biological behaviors of tumors [8, 24]. To investigate the metabolic alterations underlying 5-FU resistance of CRC, we assessed the metabolism of two important energy substrates, glucose and lactate.

**Fig. 2:**
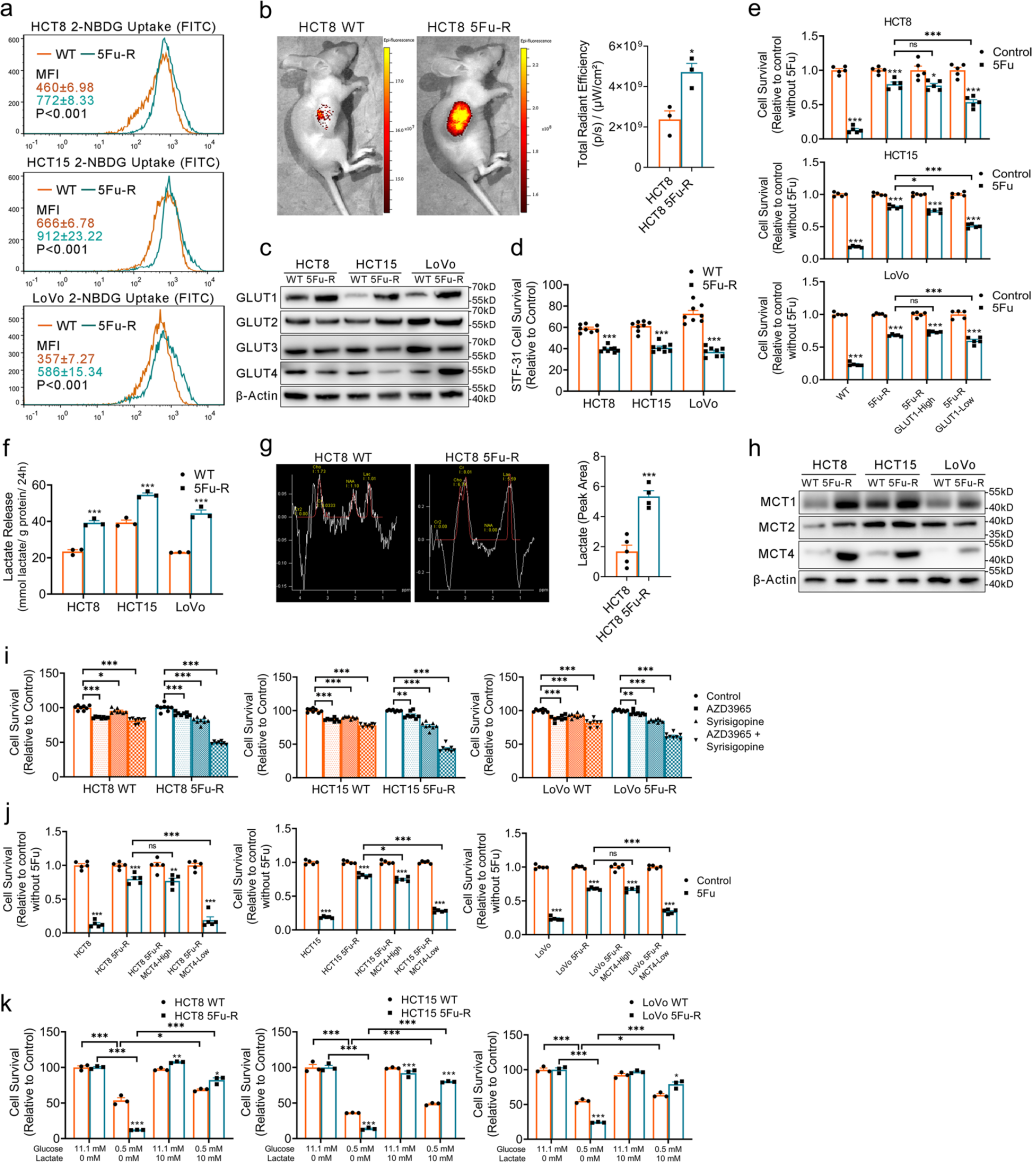
Increased glucose and lactate utilization fuels 5-FU resistance in 5-FU-R CRC cells. **a** 2-NBDG uptake by 5-FU-R and WT cells. Results are for mean MFI (FITC channel) ± SEM. **b** Tumor-bearing mice were injected with 10 nmol IRDye 800CW 2-DG Optical Probe via the tail vein and imaged 24 h later to determine in vivo glucose uptake. **c** Western blots of GLUTs in WT and 5-FU-R CRC cells. β-Actin was used as the internal reference. **d** Cell viability was measured by CCK8 assays on treatment with 10 μM STF-31 for 48 h. **e** Relative 5-FU sensitivity of 5-FU-R CRC cells flow-sorted for high and low membrane GLUT1 expression, compared with unsorted WT and 5-FU-R CRC cells as determined by CCK8 assays. Cells were cultured with 10^− 5^ M 5-FU for 72 h. **f** Lactate release from 5-FU-R cells relative to WT cells detected by colorimetry. Normalized lactate release counts using the total protein concentrations. **g** Tumor lactate concentrations of WT and 5-FU-R cell-bearing nude mice were quantified with MRS. Lactate resonance appears at 1.33 ppm. **h** Western blots of MCTs in WT and 5-FU-R cells. β-Actin was used as the internal reference. **i** Cell viability was measured by CCK8 assays on treatment with 0.5 μM AZD3965 or 1 μM Syrosingopine or both together for 72 h. **j** Relative 5-FU sensitivity of 5-FU-R CRC cells flow-sorted for high and low membrane MCT4 expression, compared with unsorted WT and 5-FU-R CRC cells as determined by CCK8 assays. Cell were cultured with 10^− 5^ M 5-FU for 72 h. **k** Effect of glucose deprivation or exogenous lactate supplementation on the survival of WT and 5-FU-R cells. Different concentrations of glucose and lactate were added to the culture medium for 48 h. WT or 5-FU-R cells cultured in normal complete RPMI-1640 medium were used as a baseline. For all studies *n* ≥ 3. Data are presented as means ± SEM. Bar chart data were compared by Student’s t-test or ANOVA (ns = not significant, * *p <* 0.05, ** *p <* 0.01, and *** *p <* 0.001)

Although tumor cells are flexible in energy sources, glucose is the primary carbon source [6, 25]. High metabolic activity is often accompanied by high glucose uptake [26]. We evaluated uptake of the fluorescence-labeled glucose analogue 2-NBDG and found that 5-FU-R cells displayed significantly increased 2-NBDG uptake (Fig. 2a). We also observed increased fluorescence of the IRDye 800CW 2-DG optical probe in the subcutaneously implanted 5-FU-R cell model compared with the WT model (Fig. 2b). Glucose uptake changes might be attributed to expression changes of glucose transporters (GLUTs). Among GLUT1-4, GLUT1 was the most up-regulated in 5-FU-R cells (Fig. 2c and Fig. S2a). We then cultured cells with STF-31 [27] (a highly selective GLUT1 inhibitor, 10 μM) for 48 h. The 5-FU-R cells were more sensitive to STF-31 compared with WT cells (Fig. 2d). To assess the relevance between 5-FU resistance and glucose uptake, we isolated 5-FU-R cells with high and low GLUT1 expression by flow cytometry. We found that lower GLUT1 expression tended to indicate a higher 5-FU sensitivity (Fig. 2e).

Enhanced aerobic glycolysis, known as the Warburg effect, is a major metabolic phenotype in tumor cells [28]. Warburg effect is presenting as glucose addiction, and additional lactate flux was also observed. Here we offer a new perspective on lactate using the most recently reported literature: Tumor cells can utilize lactate as an energy substrate, and it is definitely not a waste end product of glycolysis. Therefore, we evaluated lactate metabolism in 5-FU-R cells by conducting intracellular lactate (Fig. S2b) and lactate release assays (Fig. 2f). The 5-FU-R cells displayed significantly increased lactate release in comparison with WT cells, while there were no consistent changes in intracellular lactate. Magnetic resonance spectroscopy demonstrated higher lactate concentration in the subcutaneously implanted tumors formed by 5-FU-R cells (Fig. 2g). To further explore the characteristics and regulation of lactate metabolism in 5-FU-R cells, the expression levels of monocarboxylic acid transporters (MCTs) were examined. We observed significant up-regulation of MCT1 and MCT4 in 5-FU-R cells (Fig. 2h and Fig. S2c). We then treated cells with AZD3965 [29] (a highly selective MCT1 inhibitor) and syrosingopine [30] (a MCT inhibitor, with far higher affinity for MCT4) alone and in combination for 72 h. All CRC WT and resistant cell lines were susceptible to syrosingopine. The 5-FU-R cells displayed higher sensitivity to syrosingopine compared with WT cells, and syrosingopine combined with AZD3965 exhibited higher activity in 5-FU-R cells compared with syrosingopine alone or AZD3965 alone (Fig. 2i). These results indicated that MCTs, in particular MCT4, are important for remodeling of metabolism in 5-FU-R cells. We performed flow sorting as described above, and a significantly increased 5-FU sensitivity was observed in 5-FU-R cells with low MCT4 expression (Fig. 2j).

Lactate dehydrogenase (LDH) is a main metabolic enzyme for glycolysis and lactate production [31]. Notably, 5-FU-R cells showed high LDH expression (Fig. S2d) and intracellular LDH catalytic activity was significantly enhanced (Fig. S2e). Full oxidation of glucose to CO_2_ has been thought to provide energy to most cells, and lactate is a by-product of incomplete oxidation. Recent studies showed lactate can serve as an important carbon source under aerobic conditions, and intracellular lactate oxidation occurs in the mitochondria [32, 33]. These findings contradict our observations of mitochondrial damage and increased lactate flux in 5-FU-R cells at the same time. We thus examined the distribution of mitochondria and LDHA in cells. Despite the lower mitochondrial content in 5-FU-R cells, LDHA strongly co-localized with mitochondria (Fig. S2f). This finding suggests that mitochondria remain well lactate oxygenated under mitochondrial injury in 5-FU-R cells. Hence, our results indicate that MCTs and LDH mediated lactate exchange between the inside and outside of cells and maintain relatively constant lactate levels in 5-FU-R cells.

To analyze the dependence of 5-FU responsiveness on glucose and lactate metabolism, we cultured 5-FU-R and WT cell lines under low glucose (0.5 mM glucose) or exogenous lactate supplementation conditions (10 mM lactate) for 48 h. CRC cells were dependent on adequate glucose for viability, and 5-FU-R cells exhibited a more severe glucose addiction. When external glucose supplies are insufficient, lactate is more likely to become the alternative carbon source for cell survival in 5-FU-R cells (Fig. 2k). These results suggest that 5-FU-R cells have an increased dependence on glucose and lactate utilization, and the choice of energy substrates becomes much more flexible. Additionally, this metabolic plasticity is associated with sensitivity or resistance of 5-FU treatment.

### Glycolysis and PPP are alternative pathways of energy supply in 5-FU-R CRC cells

**Fig. 3:**
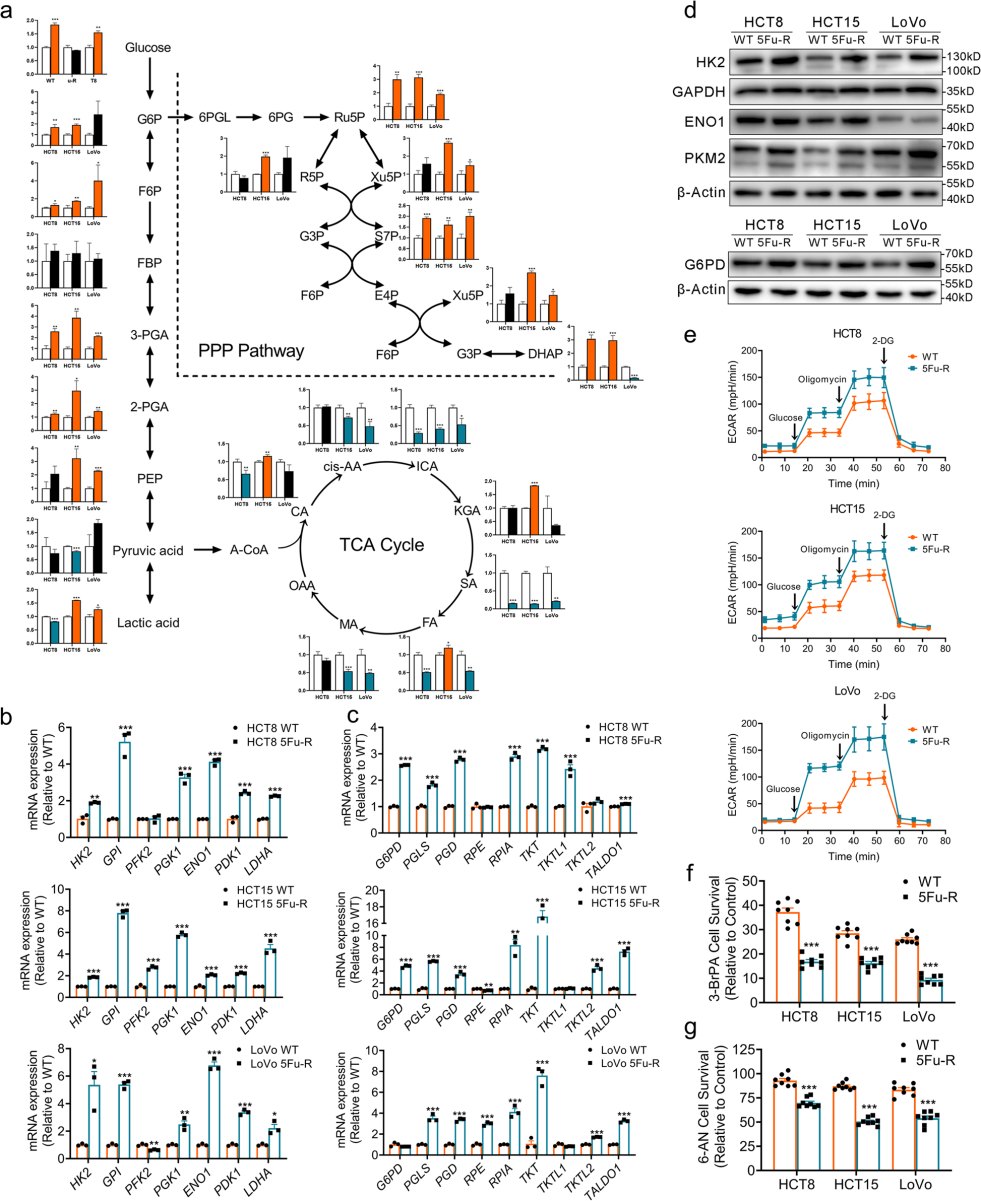
Increased glycolysis and PPP in 5-FU-R CRC cells. **a** Major metabolites altered in the glycolytic pathway, the PPP pathway, and the TCA cycle in 5-FU-R CRC cells compared with WT CRC cells. **b** RT-qPCR analysis of expression of key glycolytic enzyme genes in 5-FU-R cells relative to WT cells. *ACTB* was used as the internal reference. **c** RT-qPCR analysis of expression of genes for key enzymes of the PPP pathway in 5-FU-R cells relative to WT cells. *ACTB* was used as the internal reference. **d** Western blots of key glycolytic enzymes and G6PD in WT and 5-FU-R CRC cells. β-Actin was used as the internal reference. **e** 5-FU-R and WT cells were distributed into 96-well Seahorse assay plates and glucose (100 mM), oligomycin (10 μM) and 2-DG (500 mM) were successively added to measure ECAR. **f** Cell viability was measured by CCK8 assays on treatment with 50 μM 3-BrPA for 48 h. WT or 5-FU-R cells cultured without 3-BrPA were used as a baseline. **g** Cell viability was measured by CCK8 assays on treatment with 0.5 μM 6-AN for 48 h. WT or 5-FU-R cells cultured without 6-AN were used as a baseline. For all studies n ≥ 3. Data are presented as means ± SEM. Bar chart data were compared by Student’s-t test (* *p <* 0.05, ** *p <* 0.01, and *** *p <* 0.001)

The detailed glucose metabolic profiles in 5-FU-R cells were investigated using UHPLC-MS/MS followed by unsupervised hierarchical clustering. UHPLC-MS/MS analysis indicated differential metabolites of central carbon metabolism in 5-FU-R cells compared with WT cells (Fig. S3a). Analysis of intermediate metabolites of glucose metabolite pools revealed increased fluxes of glycolysis and PPP in 5-FU-R cells compared with WT cells (Fig. 3a). Metabolic activities are often determined by the metabolic enzymes. Thus, we examined the mRNA and protein expressions of enzymes involved in glucose metabolism. Both mRNA and protein levels of enzymes involved in glycolysis and PPP including hexokinase 2 (HK2), enolase 1 (ENO1), pyruvate kinase M2 (PKM2), and glucose-6-phosphate dehydrogenase (G6PD) were up-regulated in 5-FU-R cells compared with WT cells (Fig. 3b-d). Hence, we speculated that glycolysis and the PPP are activated as a compensatory mechanism for the impaired mitochondrial respiration to ensure energy supply in 5-FU-R cells. This may also explain the increased fluxes of glucose and lactate in 5-FU-R cells.

We also determined the ECAR to assess glycolytic stress and found that 5-FU-R cells showed a trend toward a higher ECAR (Fig. 3e). Increased basal glycolysis and glycolytic capacity were observed, and the glycolytic reserve was markedly diminished in 5-FU-R cells (Fig. S3b). To determine whether glycolysis and PPP are critical for 5-FU-R cell survival, we cultured cells with 3-bromopyruvatic acid [34] (a selective glycolysis inhibitor) or 6-aminonicotinamide [35] (a competitive inhibitor of G6PD) for 48 h. Despite the abundance of oxygen, 5-FU-R cells relied heavily on glycolysis and PPP pathways for survival (Fig. 3f, g).

These results indicate that a conversion to glycolysis and PPP occurs in the metabolic reprogramming of 5-FU-R CRC cells. Prolonged exposure to 5-FU leads to progressive irreversible mitochondrial damage in CRC cells. Metabolic reprogramming is driven by mitochondrial dysfunction and damage, and the adaptive augmented glycolysis and PPP might be associated with 5-FU resistance.

### HIF-1α is a 5-FU resistance and prognostic biomarker in CRC patients

**Fig. 4:**
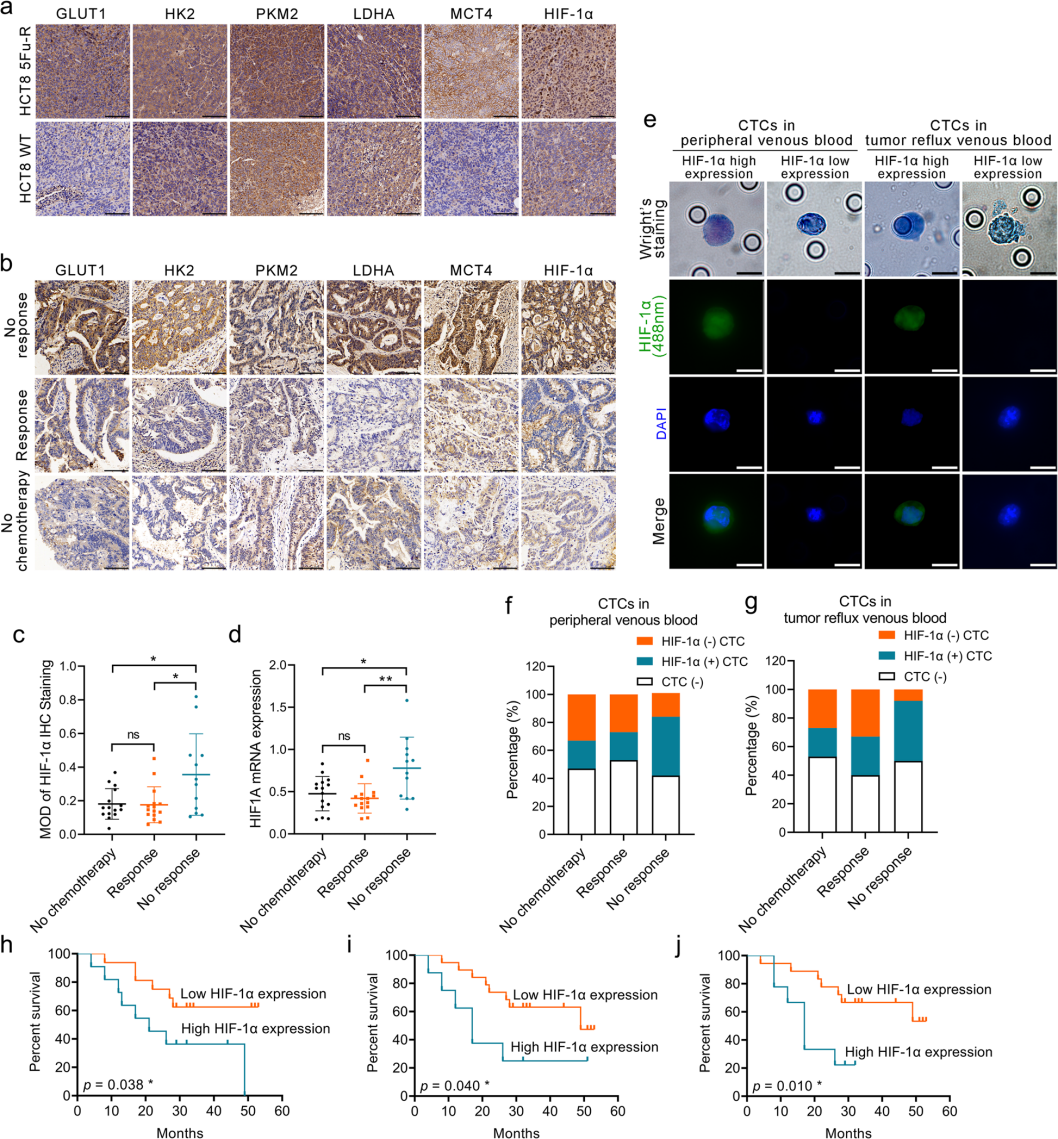
HIF-1α is a 5-FU resistance and prognostic biomarker in CRC patients. **a** Images of IHC staining for GLUT1, HK2, PKM2, LDHA, MCT4, and HIF-1α on tumor sections in subcutaneous transplantation tumor models. Scale bar = 100 μm. **b** Images of IHC staining for GLUT1, HK2, PKM2, LDHA, MCT4, and HIF-1α on tumor sections in CRC patients. Scale bar = 100 μm. **c** Comparison of IHC staining for HIF-1α in CRC patients. The grayscale HIF-1α signal is the mean optical density (MOD) of staining. **d** Comparison of *HIF1A* mRNA expression in CRC patients as assessed by RT-qPCR. *ACTB* was used as the internal reference. **e** Representative images of Wright’s staining and HIF-1α immunofluorescence staining (green) of CTCs in the blood of peripheral veins and tumor reflux veins. Scale bar = 10 μm. **f** Histograms illustrating the detection of CTCs and the proportions of HIF-1α-positive CTCs in peripheral venous blood. **g** Histograms illustrating the detection of CTCs and the proportions of HIF-1α-positive CTCs in tumor reflux venous blood. **h** Kaplan-Meier estimates of DFS of CRC patients on fluorouracil analog-based chemotherapy with high (MOD ≥0.2, *n* = 11) or low (MOD < 0.2, *n* = 16) HIF-1α expression as assessed by the intensity of the grayscale signal of IHC images. **i** Kaplan-Meier estimates of DFS of CRC patients on fluorouracil analog-based chemotherapy who had either HIF-1α-positive CTCs (*n* = 8) or HIF-1α-negative CTCs (*n* = 19) in peripheral venous blood. **j** Kaplan-Meier estimates of DFS of CRC patients on fluorouracil analog-based chemotherapy who had either HIF-1α-positive CTCs (*n* = 9) or HIF-1α-negative CTCs (*n* = 18) in tumor reflux venous blood. Bar chart data were compared by ANOVA (ns = not significant, * *p <* 0.05, and ** *p <* 0.01), and results are shown as means ± SEM. Survival was compared by log rank (Mantel-Cox) testing

We next examined whether metabolic reprogramming of glucose is involved in the pathogenesis of 5-FU resistance in CRC in both animal models and human. We detected the expressions of glycolytic enzymes and membrane transporters, including GLUT1, HK2, PKM2, LDHA, and MCT4, in subcutaneous transplantation tumor models from WT and 5-FU-R HCT8 cells by IHC. We also examined three groups of Dukes’ C or D stage CRC patients: the no chemotherapy group (without preoperative chemotherapy), response group (good response to preoperative fluorouracil analog–based chemotherapy), and no response group (poor response to preoperative fluorouracil analog–based chemotherapy) (Table S1). The expressions of glycolytic enzymes and membrane transporters were assessed in primary tumors of the three CRC patient groups using IHC. Consistent with the in vitro 5-FU-R CRC cell lines, the protein levels were elevated in both 5-FU-R HCT8 tumors (Fig. 4a) and the no response CRC patient group (Fig. 4b). 5-FU-R CRC cells displayed high glycolysis even compared with WT CRC cells that already show increased glycolysis.

Increased aerobic glycolysis has been associated with the acquisition of a number of tumor phenotypes, including chemotherapy resistance. We further examined the mechanisms of 5-FU resistance by investigating glycolysis regulation. The HIF-1α transcription factor is a master glycolytic activity regulator that upregulates genes encoding glycolytic enzymes and membrane transporters [13, 14]. We observed significantly higher levels of HIF-1α in 5-FU-R HCT8 transplantation tumors compared with WT models (Fig. 4a). The CRC no response patient group demonstrated a markedly increased HIF-1α protein and mRNA expression compared with the other two groups (Fig. 4b-d).

We further examined CTCs in the blood of peripheral veins and tumor reflux veins (such as the inferior mesenteric vein in rectal cancer) in the three CRC patient groups (Fig. 4e). The detection rate of CTCs did not differ significantly among the three groups, and the no response group had higher proportions of HIF-1α-positive CTCs both in peripheral venous blood and tumor reflux venous blood compared with the other two groups (Fig. 4f, g). These results illustrated that resistance of CRC to fluorouracil analog was associated with increased expression of HIF-1α in tumor cells.

Furthermore, CRC patients subjected to preoperative fluorouracil analog–based chemotherapy with high HIF-1α expression in primary tumors or CTCs demonstrated a poor disease-free survival after surgery (Fig. 4h-j). Recent studies showed that CTCs are a strong independent prognostic factor for progression-free survival and overall survival of CRC patients [36, 37]. We found significant correlations between HIF-1α expression of CTCs and disease-free survival in patients previously treated with fluorouracil analog, especially CTCs in the reflux veins of tumor (Fig. 4j).

We examined whether *HIF1A* mRNA levels showed predictive value for 5-FU chemotherapeutic efficacy using the public Gene Expression Omnibus databases (GSE69657, GSE104645). The results revealed no differences in *HIF1A* expression in primary tumors before chemotherapy between patients who responded and those who did not respond to fluorouracil analog–based chemotherapy (Fig. S4a). This observation suggested that high HIF-1α expression is a result from long-term exposure to fluorouracil analog (5-FU or Capecitabine) in CRC patients with acquired resistance and HIF-1α expression in primary tumors before exposure to fluorouracil analog may not have a predictive value for chemotherapy efficacy.

These results indicate that failure of fluorouracil analog-based chemotherapy in CRC is accompanied by increased HIF-1α levels. Thus, HIF-1α may serve as a potential biomarker for assessment and prognosis in CRC patients with fluorouracil analog treatment.

### HIF-1α is a master regulator of 5-FU resistance and glucose metabolic reprogramming in 5-FU-R CRC cells

**Fig. 5:**
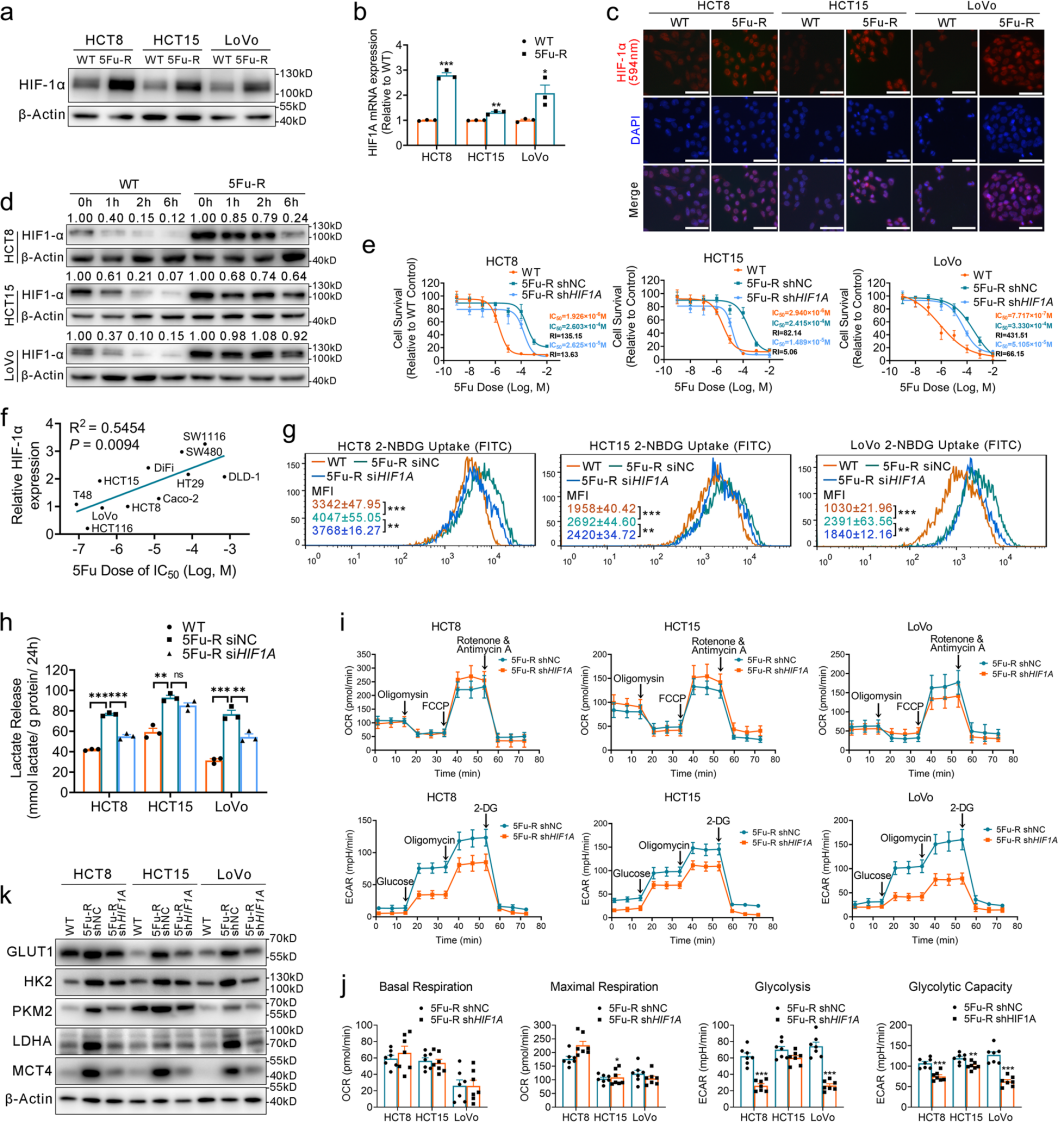
HIF-1α is a master regulator of 5-FU resistance and glucose metabolic reprogramming in 5-FU-R CRC cells. **a** Western blots of HIF-1α in WT and 5-FU-R cells. β-Actin was used as the internal reference. **b** RT-qPCR analysis of *HIF1A* gene expression in 5-FU-R cells relative to WT cells. *ACTB* was used as the internal reference. **c** Immunocytofluorescence staining of HIF-1α (red). Merged images represent overlays of HIF-1α and nuclear staining in blue by DAPI. Scale bar = 20 μm. **d** WT and 5-FU-R cells cultured with 100 μM CHX for 1 h, 2 h, and 6 h to compare the stability of HIF-1α. β-Actin was used as the internal reference. **e** Effect of *HIF1A* knock-down on 5-FU sensitivity of 5-FU-R CRC cells. Viability was measured by CCK8 assay after treating cells with increasing concentrations of 5-FU for 72 h. **f** Correlations between HIF-1α expression and IC_50_ of 5-FU in 11 CRC cell lines by Pearson correlation analysis. **g** Effect of *HIF1A* knock-down on 2-NBDG uptake by 5-FU-R CRC cells. **h** Effect of *HIF1A* knock-down on lactate release from 5-FU-R CRC cells. Normalized lactate release counts using the total protein concentrations. **i** Measurement of OCR and ECAR of *HIF1A* knock-down 5-FU-R cells and control cells. **j** Comparison of basal respiration, maximal respiration, glycolysis and glycolytic capacity in *HIF1A* knock-down and control 5-FU-R CRC cells. **k** Effect of *HIF1A* knock-down on expression of the key glycolytic enzymes GLUT1 and MCT4. β-Actin was used as the internal reference. For all studies *n* ≥ 3. Data are means ± SEM. Bar chart data were compared by Student’s t-test or ANOVA (ns = not significant, * *p <* 0.05, ** *p <* 0.01, and *** *p <* 0.001). R^2^ denotes the Pearson correlation coefficient and the *P* value indicates the significance of the correlation

We further evaluated the functional role of HIF-1α in 5-FU-R CRC cells. The 5-FU-R cells express elevated HIF-1α protein and mRNA levels under normoxia (Fig. 5a, b). Immunofluorescence showed that HIF-1α localized in the cytoplasm and nucleus in WT cells, whereas HIF-1α expression was strongly nuclear in 5-FU-R cells (Fig. 5c). Western blot also showed very high expressions of nuclear HIF-1α in 5-FU-R cells (Fig. S4b).

Tumor cells in vitro and in vivo show a notable difference, as tumor cells are exposed to a complex oxygen microenvironment in vivo. We observed that HIF-1α expression in 5-FU-R cells showed increased responses to 1% oxygen (Fig. S4c), suggesting that the high HIF-1α expression in 5-FU-R cells is independent of oxygen concentration. HIF-1α is readily degraded under normoxia conditions [13]. We performed experiments with cycloheximide, a protein synthesis inhibitor, and found that HIF-1α was degraded quickly in WT cells in normoxia, whereas it was much more stable in 5-FU-R cells (Fig. 5d). Taken together, these results show that HIF-1α protein is nuclear and stabilized in 5-FU-R cells.

To further examine the effects of HIF-1α on 5-FU resistance and glucose metabolism in 5-FU-R cells, we stably knocked down endogenous HIF-1α using lentivirally-expressed *HIF1A* shRNA (sh*HIF1A*) (Fig. S4d). *HIF1A* knock-down partially reversed 5-FU resistance in the 5-FU-R cell lines (Fig. 5e). We investigated HIF-1α protein levels in 11 CRC cell lines and correlated them with the IC_50_ of 5-FU (R^2^ = 0.5454, *P* = 0.0094) (Fig. 5f and Fig. S4e, f); 5-FU resistance showed a direct correlation with HIF-1α expression. Based on this, it is well-documented that HIF-1α expression level can affect 5-FU sensitivity in CRC cells.

We then evaluated the influence of HIF-1α on glucose metabolism in 5-FU-R cells. *HIF1A* knock-down in 5-FU-R cells resulted in significantly decreased 2-NBDG uptake and lactate release compared with control cells (Fig. 5g, h). *HIF1A* knock-down decreased glycolysis and glycolytic capacity as detected by OCR and ECAR, but showed no significant effect on mitochondrial respiration (Fig. 5i, j). HIF-1α downstream glycolytic enzymes and transporters including GLUT1, HK2, PKM2, LDHA and MCT4 were down-regulated in sh*HIF1A* 5-FU-R cells (Fig. 5k). These results suggest that HIF-1α mediates 5-FU resistance by glucose metabolic reprogramming.

### Both *HIF1A* knock-down and pharmacological inhibition of HIF-1α improve 5-FU resistance in vivo

**Fig. 6:**
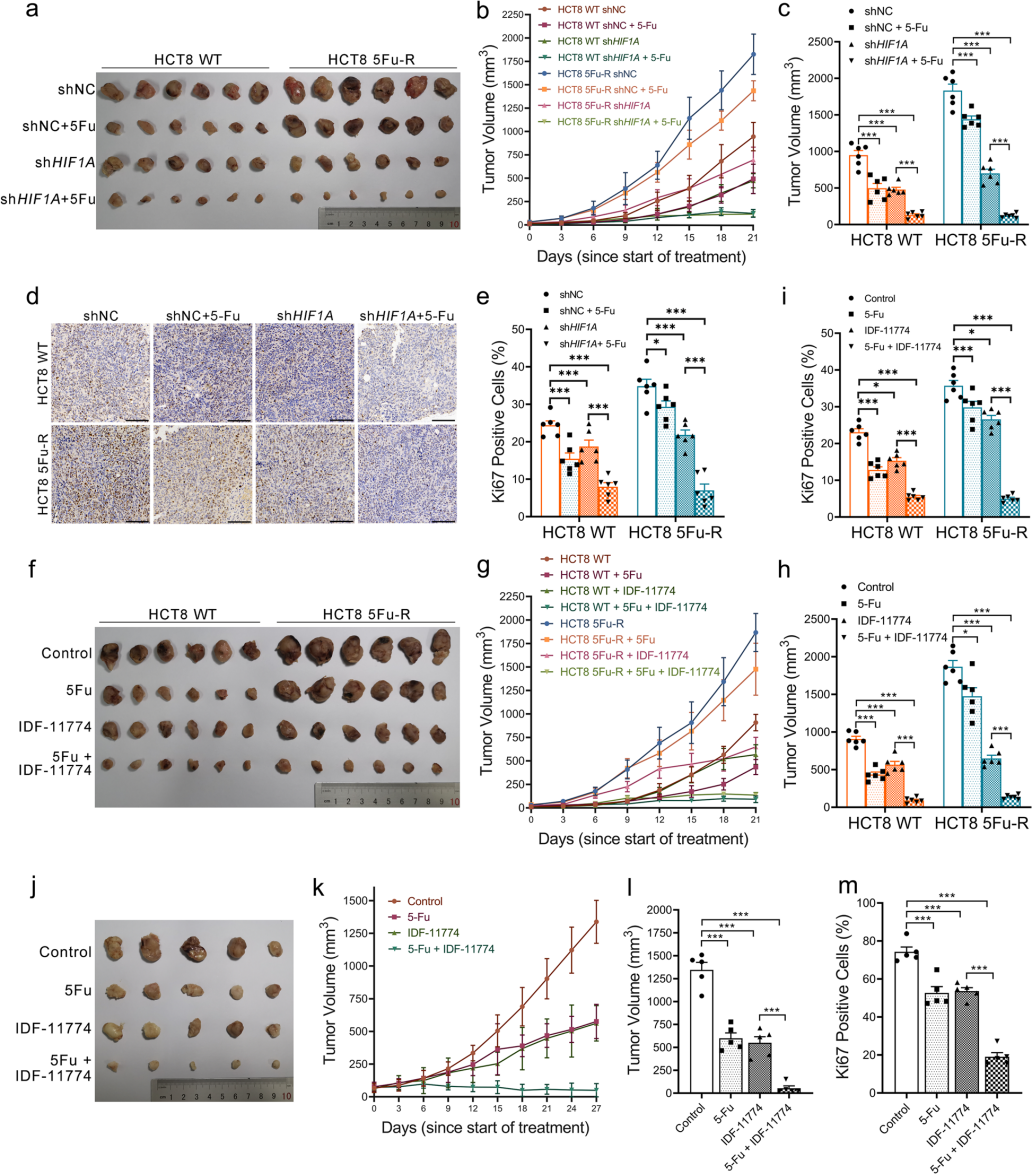
Both *HIF1A* knock-down and pharmacological inhibition of HIF-1α are effective for reducing 5-FU resistance in vivo. **a-e** Effect of *HIF1A* knockout on 5-FU resistance in subcutaneously-implanted WT or 5-FU-R cells in a nude mouse model. One week after subcutaneous injection, the mice were treated intraperitoneally with 25 mg/kg 5-FU or saline three times a week. Tumors were harvested when the diameter was > 1.5 cm (**a**). Tumor volumes were quantified by *V* = *L* × *W*^2^/2 (where *L* is the length and *W* is the width) (**b**, **c**). Representative images of IHC staining for Ki-67, scale bar = 200 μm (**d**). Percentage of Ki-67-positive cells (**e**). **f-i** Effect of IDF-11774 on 5-FU resistance in subcutaneously-implanted WT or 5-FU-R cells in a nude mouse model. Tumors harvested from subcutaneously-implanted nude mice treated with saline (control), 5-FU alone (25 mg/kg, three times a week), IDF-11774 alone (30 mg/kg, twice a week) or 5-FU together with IDF-11774 (**f**). Tumor volumes were quantified by *V* = *L* × *W*^2^/2 (where *L* is the length and *W* is the width) (**g**, **h**). Percentage of Ki-67-positive cells (**i**). **j-m** Effect of IDF-11774 on 5-FU resistance in a PDXs NOD/scid mouse model. Tumors harvested from subcutaneously-implanted nude mice treated with saline (control), 5-FU alone (25 mg/kg, three times a week), IDF-11774 alone (30 mg/kg, twice a week) or 5-FU together with IDF-11774 (**j**). Tumor volumes were quantified by *V* = *L* × *W*^2^/2 (where *L* is the length and *W* is the width) (**k**, **l**). Percentage of Ki-67-positive cells (**m**). Data are presented as means ± SEM. Bar chart data were compared by ANOVA (* *p <* 0.05, ** *p <* 0.01, and *** *p <* 0.001)

To evaluate the influence of HIF-1α on 5-FU resistance of CRC cells in vivo environments, we established subcutaneous transplantation tumor models of WT and 5-FU-R HCT8 cells with or without stable *HIF1A* knock-down in nude mice. Tumor-bearing mice were treated with 5-FU or saline one week after implantation. We observed significant reduction in tumor volume and proliferation in 5-FU-treated WT HCT8-bearing mice, with no change in body weight. However, 5-FU failed to show effective antitumor efficacy in 5-FU-R HCT8-bearing mice. *HIF1A* knock-down slowed down tumor growth and diminished the proportion of Ki-67-positive cells in both WT and 5-FU-R groups. Tumor volume and proliferation of *HIF1A* knock-down tumors decreased significantly upon 5-FU treatment in both WT and 5-FU-R groups (Fig. 6a-e and Fig. S5b). knock-down of *HIF1A* caused a significant decrease in the expression of glycolytic enzymes and membrane transporters, including HIF-1α, GLUT1, HK2, PKM2, LDHA, and MCT4, in IHC (Fig. S5a). There were no significant changes in protein expressions after short-term 5-FU treatment compared with saline treatment (Fig. S5a).

To demonstrate the potential clinical value of HIF-1α as an intervention, we investigated whether pharmacological inhibition of HIF-1α in vivo had the same effect as *HIF1A* knock-down. We injected 5-FU, the HIF-1α inhibitor IDF-11774 [38] or saline in subcutaneous tumor models in nude mice starting one week after implantation. Tumor volume and proliferation were inhibited by IDF-11774 to the same extent as *HIF1A* knock-down in both WT and 5-FU-R groups (Fig. 6f-i and Fig. S5c, d). To further establish the value of pharmacological inhibition of HIF-1α in increasing 5-FU sensitivity, we selected a patient with rectal cancer (male, Dukes’ C) who did not respond to 5-FU-based preoperative neoadjuvant chemotherapy. We generated patient-derived xenografts in nude mice to mimic actual clinical presentation. The combination of 5-FU in combination with IDF-11774 markedly inhibited tumor growth and proliferation compared with 5-FU alone (Fig. 6j-m and Fig. S5e, f).

These findings indicate that HIF-1α gene knock-down and pharmacological inhibition are effective treatments for 5-FU-R CRC in vivo.

### ROS-induced HIF-1α high expression and 5-FU resistance via the activated PI3K/Akt pathway in 5-FU-R CRC cells

**Fig. 7:**
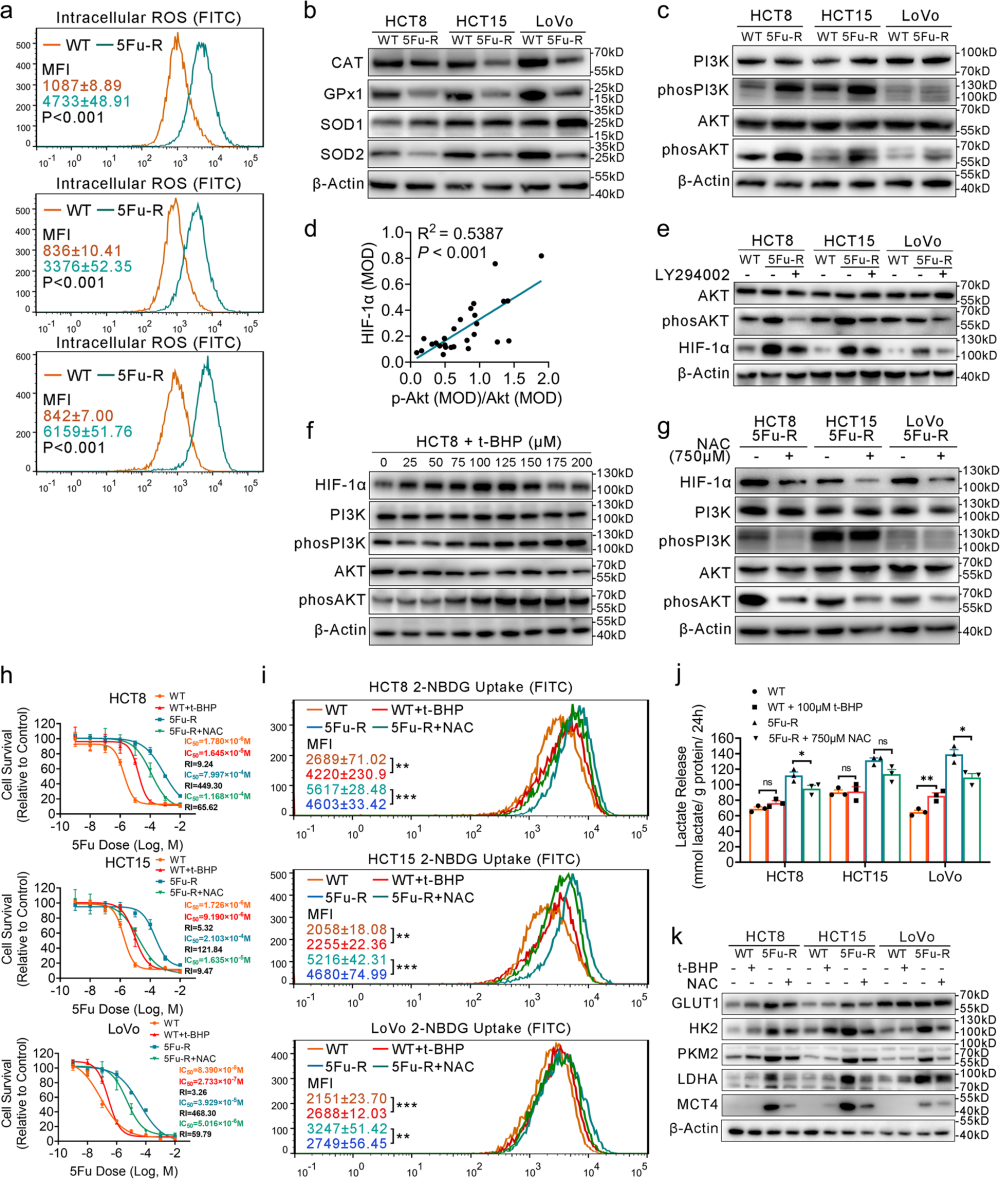
ROS/PI3K/AKT pathway activation boosts HIF-1α levels and induces 5-FU resistance. **a** ROS levels in 5-FU-R and WT cells. Results shown as mean MFI (FITC channel) ± SEM. **b** Western blots of ROS scavenging enzymes. β-Actin was used as the internal reference. **c** Western blots of PI3K/AKT pathway members from WT and 5-FU-R CRC cells. β-Actin was used as the internal reference. **d** Correlations between levels of p-Akt/Akt and HIF-1α proteins by Pearson correlation analysis. **e** 5-FU-R cells were treated with 25 μM LY294002 for 48 h. Western blots of HIF-1α, AKT, and p-AKT. β-Actin was used as the internal reference. **f** HCT8 (WT) cells were treated with titrated concentrations of t-BHP (0 μM to 200 μM) for 4 h. Western blots of HIF-1α and PI3K/AKT pathway members. β-Actin was used as the internal reference. **g** 5-FU-R cells were treated with 750 μM NAC for 48 h. Western blots of HIF-1α and PI3K/AKT pathway members. β-Actin was used as the internal reference. **h** Effect on 5-FU sensitivity as determined by CCK8 assays. WT cells were treated with t-BHP (100 μM, 4 h) and 5-FU-R cells were treated with NAC (750 μM, 48 h). Cells were cultured with increasing concentrations of 5-FU for 72 h after t-BHP or NCA treatment. **i** Effect of modulating ROS levels on 2-NBDG uptake. **j** Effect of modulating ROS levels on lactate release. Normalized lactate release counts using the total protein concentrations. **k** Effect of modulating ROS levels on expression of the key glycolytic enzymes GLUT1 and MCT4. β-Actin was used as the internal reference. For all studies n ≥ 3. Data are presented as means ± SEM. Data were analyzed by Student’s t-test or ANOVA (ns = not significant, * *p <* 0.05, ** *p <* 0.01, and *** *p <* 0.001). R^2^ denotes the Pearson correlation coefficient and the *P* value indicates the significance of the correlation

ROS plays an important role in tumorigenesis and tumor development [39]. ROS is linked with damaged and dysfunctional mitochondria [40], and the release of NADPH from oxidative PPP counteracts the excess ROS and helps maintain intracellular ROS balance [41]. These phenomena are in line with the observed mitochondrial damage and high levels of PPP in 5-FU-R cells, which suggests that 5-FU-R cells are in the high ROS load condition. We measured ROS levels by flow cytometry and found high ROS levels in 5-FU-R cells (Fig. 7a). We next considered whether impairment of ROS clearance was associated with 5-FU resistance. The expressions and activities of mitochondrial ROS-scavenging enzymes catalase (CAT), glutathione peroxidase (GPx), and superoxide dimutase 2 (SOD2) were significantly decreased in 5-FU-R cells (Fig. 7b and Fig. S6a, b), while the changes in cytoplasmic ROS-scavenging enzyme superoxide dimutase 1 (SOD1) were inconsistent among the different cell lines (Fig. 7b). Taken together, these data supported that both the increased generation of ROS and the destruction of mitochondrial ROS-scavenging enzyme system induced the accumulation of ROS in 5-FU-R cells in two different aspects.

The PI3K/Akt pathway plays a central role in the regulation of substance and energy metabolism [42]. Up-regulated HIF-1α expression and increased glycolysis in tumor cells are dependent on the activated PI3K/Akt pathway, regardless of oxygen levels [43]. We hypothesized that the PI3K/Akt pathway may promote HIF-1α-mediated glucose metabolic reprogramming and 5-FU resistance in 5-FU-resistant CRC. Western blot showed that phosphorylated PI3K (p-PI3K) and phosphorylated Akt (p-Akt) were up-regulated in 5-FU-R cells (Fig. 7c). In addition, IHC revealed p-Akt and HIF-1α expression in tumors of 27 CRC patients who received preoperative fluorouracil analog–based chemotherapy (Fig. S6c). Pearson correlation analysis showed that p-Akt/Akt was significantly positively correlated with HIF-1α (R^2^ = 0.5387, *P* < 0.001) (Fig. 7d). Furthermore, the level of p-Akt dramatically decreased in 5-FU-R cells upon treatment with LY294002 [44] (a broad-spectrum inhibitor of PI3K) for 48 h, and HIF-1α protein and mRNA levels were decreased (Fig. 7e and Fig. S6d). Our results indicated that the activated PI3K/Akt pathway up-regulates HIF-1α in 5-FU-R cells. However, significant changes of phosphorylation levels of PI3K and Akt were not observed in 5-FU-R cells with *HIF1A* knock-down (Fig. S6e), suggesting that there is no feedback regulation between HIF-1α and the PI3K/Akt pathway.

Previous studies revealed that ROS directly activates PI3K to amplify its downstream signaling through reversible inactivation of the phosphatase and tensin homolog (PTEN) [45, 46]. Other reports established a reciprocal regulation of ROS and HIF-1α by complex interactions [17–19]. The regulatory circuit between ROS and HIF-1α remains elusive due to the vast heterogeneity of tumors. We hypothesized that ROS-induced HIF-1α high expression in 5-FU-R CRC cells is mediated by the activated PI3K/Akt signaling pathway. To investigate the effects of ROS on the PI3K/Akt pathway and HIF-1α expression in 5-FU resistance, we generated cell models with decreased and increased levels of intracellular ROS. HIF-1α, p-PI3K, and p-Akt gradually increased in response to tert-butylhydroperoxide (t-BHP) from 0 μM to 100 μM in cultured WT HCT8 cells (Fig. 7f). Moreover, we detected decreased expression of HIF-1α, p-PI3K, and p-Akt in 5-FU-R cells treated with N-acetylcysteine (NAC, a ROS scavenger) (Fig. 7g). Thus, both PI3K/Akt pathway activity and HIF-1α expression were influenced by ROS. Collectively, these results suggested that ROS-induced HIF-1α high expression in 5-FU-R CRC cells is, to a certain extent, mediated via PI3K/Akt pathway activation.

We next verified if modulating ROS levels of CRC cells could improve 5-FU resistance and shift the glucose metabolic phenotype. Treatment with 100 μM t-BHP up-regulated ROS in WT cells, while 750 μM NAC diminished ROS in 5-FU-R cells. Thus, scavenging ROS in 5-FU-R cells can increase sensitivity to 5-FU, while accumulation of ROS in WT cells increased resistance to 5-FU (Fig. 7h). Furthermore, ROS elimination in 5-FU-R cells led to significantly decreased 2-NBDG uptake, lactate release, and expressions of glycolysis-related proteins in contrast to controls without ROS scavenger, while an enhanced glycolytic phenotype observed in WT cells cultured with t-BHP (Fig. 7i-k). Together these findings establish that the response of CRC cells to 5-FU can be controlled through modulation of intracellular ROS levels.

### The HIF-1α/β-catenin transcriptional complex alters the spatial distribution of HIF-1α and contributes to 5-FU resistance and glycolytic activity in 5-FU-R CRC cells

**Fig. 8:**
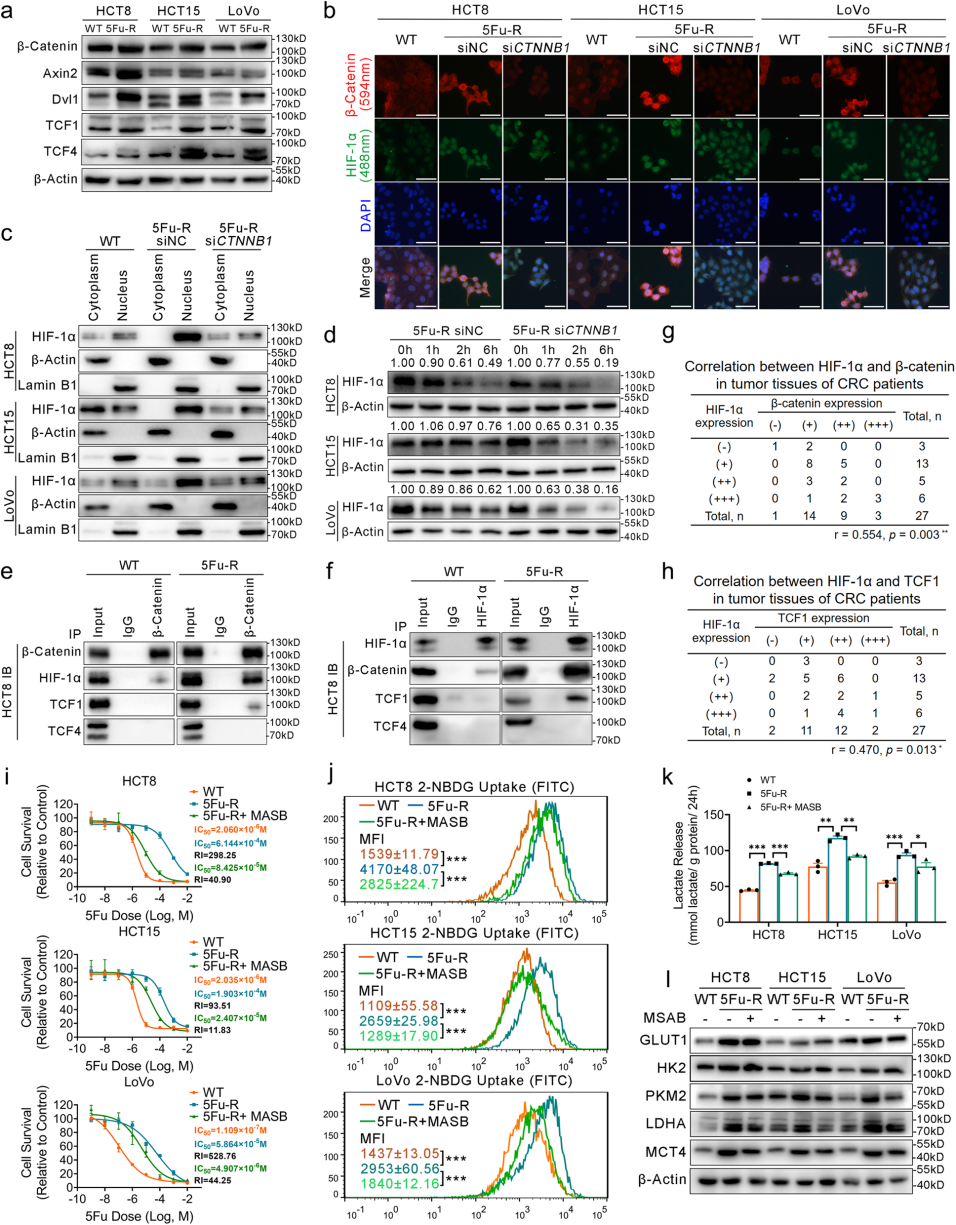
The HIF-1α/β-catenin transcriptional complex contributes to 5-FU resistance and glycolytic activity. **a** Western blots of Wnt/β-catenin signaling pathway members from WT and 5-FU-R cells. β-Actin was used as the internal reference. **b** Immunocytofluorescence staining of β-catenin and HIF-1α. Merged images show the overlap of β-catenin (red), HIF-1α (green) and nuclear staining by DAPI (blue). **c** Effect of siRNA *CTNNB1* knock-down on distribution of HIF-1α analyzed by Western blotting. Lamin B1 is a nuclear protein reference, and β-Actin is a cytoplasmic marker. **d** Effect of siRNA *CTNNB1* knock-down in 5-FU-R cells on the stability of HIF-1α as assessed by Western blotting. β-Actin was used as the internal reference. **e** Anti-β-catenin co-immunoprecipitation experiment to identify interactions between β-catenin, HIF-1α, TCF1, and TCF4 in WT and 5-FU-R HCT8 cells. **f** Anti-HIF-1α co-immunoprecipitation experiment to identify interactions between HIF-1α, β-catenin, TCF1, and TCF4 in WT and 5-FU-R HCT8 cells. **g** Correlations between levels of β-catenin and HIF-1α proteins by Spearman rank analysis. **h** Correlations between levels of TCF1 and HIF-1α proteins by Spearman rank analysis. **i** Effect of 10 μM MSAB treatment for 72 h on 5-FU sensitivity of 5-FU-R cells. Cell viability was measured by the CCK8 assay after treating with increasing concentrations of 5-FU for 72 h. **j** Effect of 10 μM MSAB treatment on 2-NBDG uptake. **k** Effect of 10 μM MSAB treatment on lactate release. Normalized lactate release counts using the total protein concentrations. **l** Effect of 10 μM MSAB treatment on expression of the key glycolytic enzymes GLUT1 and MCT4. β-Actin was used as the internal reference. For all studies n ≥ 3. Data are presented as means ± SEM. Data were analyzed by ANOVA (* *p <* 0.05, ** *p <* 0.01, and *** *p <* 0.001). *r* denotes Spearman rank correlation value and the *p* value denotes the significance level

Aberrant activation of Wnt/β-catenin signaling is a critical factor for tumorigenesis and development [47]. Previous studies showed that β-catenin, the key molecule of Wnt signaling, interplays with HIF-1α in multiple physiological and pathological processes [48], and activated Wnt signaling pathway plays a role in chemoresistance [49]. Therefore, we hypothesized that β-catenin may affect the chemosensitivity of 5-FU through its interaction with HIF-1α. Western blot confirmed activated Wnt signaling pathway in 5-FU-R cells compared with WT cells (Fig. 8a). We examined β-catenin and found that its half-life was much longer in 5-FU-R cells in cycloheximide experiments (Fig. S7a). Immunofluorescence showed that β-catenin was located in the cell membrane, cytoplasm and nucleus in WT cells, while β-catenin was predominantly localized in the nucleus in 5-FU-R cells (Fig. 8b).

We also detected co-localization of HIF-1α and β-catenin in 5-FU-R cells (Fig. 8b). To further assess the effect of β-catenin on HIF-1α, 5-FU-R cells were transfected with siRNA targeting β-catenin (si*CTNNB1*). Knock-down of *CTNNB1* caused a portion of HIF-1α protein to migrate to the cytoplasm (Fig. 8b). We also observed up-regulated cytosolic distribution and down-regulated nuclear distribution of HIF-1α in 5-FU-R cells with *CTNNB1* knock down (Fig. 8c). Moreover, we examined the stability of HIF-1α in 5-FU-R cells following knock-down of β-catenin, and HIF-1α showed a short half-life in cells with β-catenin knock-down (Fig. 8d). These results indicated that β-catenin plays important roles in stabilization and nuclear translocation of HIF-1α in 5-FU-R CRC cells.

In particular, the interaction of β-catenin with specific receptors in the nucleus was dependent on the T cell factor/lymphoid enhancer-binding factor (TCF/LEF, hereafter referred to as TCF) [50]. We next examined the interaction of HIF-1α, β-catenin and TCF. We immunoprecipitated β-catenin and HIF-1α and detected binding in both WT and 5-FU-R cells (Fig. 8e, f and Fig. S7b, c). While HIF-1α/β-catenin complex did not bind TCF1 or TCF4 in WT cells, we found that TCF1, but not TCF4, interacted with the HIF-1α/β-catenin complex in 5-FU-R cells. This may suggest TCF1 contributes to the formation of HIF-1α/β-catenin transcriptional complex in 5-FU-R cells.

We next measured the protein expressions of β-catenin, TCF1, and HIF-1α in tumors of 27 CRC patients who received preoperative fluorouracil analog–based chemotherapy by IHC (Fig. S7d). Spearman rank correlation analysis showed a significant positive correlation between IHC scores of HIF-1α and β-catenin (*r* = 0.554, *p* = 0.003) (Fig. 8g) as well as HIF-1α and TCF1 (*r* = 0.470, *p* = 0.013) (Fig. 8h). Together, this suggests that activation of the Wnt signaling pathway and β-catenin expression may play a part in HIF-1α stabilization in 5-FU-R cells.

To investigate whether the pharmacological inhibition of β-catenin affects the metabolic phenotype and 5-FU resistance in 5-FU-R cells, we cultured 5-FU-R cells with MSAB [51] (a selective inhibitor of Wnt/β-catenin signaling) to decrease the expression of β-catenin. Suppressed β-catenin resulted in significantly diminished 5-FU resistance in 5-FU-R cells (Fig. 8i). Furthermore, 2-NBDG uptake, lactate release, and expressions of glycolysis-related proteins were significantly decreased in β-catenin-suppressed 5-FU-R cells (Fig. 8j-l). These results indicate that inhibition of β-catenin leads to decreased 5-FU resistance and glycolysis in 5-FU-R cells.

## Discussion

For more than 60 years, 5-FU has been the first-line drug for both single-drug and multi-drug chemotherapy and the most effective systemic agent for managing advanced and metastatic CRC [3, 4]. However, a considerable fraction of patients still acquire 5-FU resistance [5]. Therefore, identifying strategies on how to improve the efficacy of 5-FU and reverse 5-FU resistance are important challenges in clinical practice. Several mechanisms of 5-FU resistance have been reported, including changes in the rate of drug influx or efflux [52], intra-tumor heterogeneity [52], epigenetic factors [53], tumor microenvironment [54], and 5-FU metabolic enzymes [55]. Here, we present a mechanism of 5-FU resistance in CRC by which cancer cells remodel glucose metabolism, which renders 5-FU ineffective. We present strong evidence for the use of inhibitors or gene knock-down targeting HIF-1α, a “master regulator” of glucose metabolism [14], in diminishing 5-FU resistance in CRC.

Metabolic reprogramming is a hallmark in cancer cells [6, 7]. We found that the intracellular glucose metabolic pools changed significantly in 5-FU-resistant CRC, with abnormal glucose and lactate transport and utilization. Our results also showed that metabolic reprogramming events in the process of 5-FU resistance are an exacerbation from OXPHOS to glycolysis and PPP.

One of the most important characteristics of metabolic reprogramming in tumors is increased dependency on glycolysis for energy generation [8]. Although the energy conversion efficiency of glycolysis is not as high as OXPHOS, increased glucose uptake and a higher glycolytic flux can compensate [8]. In addition to providing energy, high glycolytic flux also provides a variety of raw materials for biosynthesis [10]. Increased aerobic glycolysis contributes to tumor progression by giving cancer cells growth advantage and drug resistance phenotypes [11]. Drug resistance of CRC cells to vincristine and oxaliplatin is overcome by knocking out polypyrimidine tract binding protein 1 (PTBP1) [56], a regulator of glycolysis. HK2 catalyzes the first rate-limiting step in glucose metabolism, and 2-DG, an inhibitor of HK2, reverses drug resistance in several in vitro models [57]. Silencing PKM2 increased docetaxel accumulation and promoted anti-tumor activity in lung cancer cells [58]. Targeting MCT1 greatly enhanced the sensitivity of human osteosarcoma cells to chemotherapy [59]. These data implied that inhibition of glycolysis by key enzymes targeting the glycolytic pathway may be a potential broad-spectrum therapeutic approach for reversing drug resistance. Our results showed that knock down or pharmacological inhibition to down-regulate HIF-1α expression inhibited glycolysis and ultimately reversed 5-FU resistance, and this may be a much more effective approach than other agents because HIF-1α regulates the entire glycolytic pathway as a critical up-stream regulator.

Activation of PPP is implicated in the development of various tumors, and PPP is strongly activated in chemotherapy-resistant cells and tumor tissues [11]. We also demonstrated a higher PPP flux both oxidative and non-oxidative pathways in 5-FU-resistant CRC cells. Flux through the oxidative PPP has a stronger ability to generate NADPH [41]. Given that NADPH has been mainly associated with ROS scavenging, high levels of ROS in 5-FU-R CRC cells may drive the activation of oxidative PPP. In additional, PPP is a necessary pathway for the biosynthesis to meet metabolic demands of glycolysis-dependent cells [41]. The higher metabolic flux into the non-oxidative PPP increases nucleotide biosynthesis and causes an increase intracellular nucleotide pools. As 5-FU is a pyrimidine analog drug, it will likely be diluted by increased intracellular nucleotide concentrations. Future research should explore a method to reverse resistance in CRC to 5-FU and other fluoropyrimidine by inhibiting non-oxidative PPP related nucleotide biosynthesis.

Previous studies showed that inhibition of energy substrates relieves drug resistance [60]. Substantial glucose influx and high expression of GLUT1 ensure the supply of metabolic substrates for a high rate of glycolysis and PPP [7, 8]. This is consistent with our observations, in which activated glycolysis was accompanied by increased glucose uptake and up-regulated GLUT1 expression in 5-FU-R cells in vitro and in vivo. Furthermore, increased level of GLUT1 was related to 5-FU resistance in 5-FU-R cells. Thus, to limit the use of glucose as an energy source might have advantages as therapeutic strategies in 5-FU resistance in CRC. We also observed significantly increased lactate efflux and up-regulation of MCT1/MCT4 in 5-FU-R cells, and high expression of MCT4 was related to 5-FU resistance. Hence, targeting MCTs not only limited glycolytic flux but also reversed 5-FU resistance, which profoundly improved the cell-killing effect of 5-FU.

Although a high glycolysis rate confers 5-FU-R cells with growth and resistance advantages, it also renders cancer cells susceptible to glucose deprivation. The glucose metabolic reprogramming in vitro and in nude mice reflects the glucose consumption capacity of 5-FU-R cells but this capacity does not necessarily reflect a real condition of glucose usage in vivo. In low glucose conditions, we found that glucose deprivation was severely detrimental to 5-FU-R cells, while lactate rescued cancer cells from extremely low glucose condition. Thus, disrupting lactate balance and use in 5-FU-resistant cells or tumors could expose their vulnerability to glucose deprivation and ultimately reverse 5-FU resistance. Co-blockade of GLUT1 and MCTs for tumor cell killing and resistance reversal should be pursued in future studies.

Increased stability of HIF-1α and enhanced transcription of its downstream genes have been widely reported in a variety of tumors during malignant progression [61]. Consistently, HIF-1α which was considered to be a major orchestrator of cellular adaptation to oxygen environment, whereas the precise oxygen-dependent prolyl hydroxylases (PHDs)-mediated regulation [62]. In addition, some non-classical regulation of HIF-1α, independent of external oxygen concentrations, has been reported [63]. The oxygen environment is complicated and fluctuant in tumor tissue in vivo. We observed high expressions of HIF-1α in 5-FU-R cells in both normoxia and hypoxia conditions. In this study, we identified novel mechanisms of HIF-1α regulation in 5-FU-resistant CRC that are independent of the classical PHD-mediated oxygen-dependent pathway. The activated PI3K/Akt pathway plays an important role in human cancer [42], and activated PI3K/Akt signaling up-regulates *HIF1A* transcription and translation [43, 64]. Moreover, PI3K/Akt signal stabilizes and trans-activates HIF-1α regardless of oxygen levels [43]. We observed that ROS accumulation leads to HIF-1α up-regulation via activated PI3K/Akt signaling pathway in 5-FU-R cells. HIF-1α mRNA and protein were regulated by PI3K/Akt signaling in 5-FU-resistant CRC, in response not to oxygen conditions but to the overload of ROS.

The Wnt/β-catenin signaling pathway plays an important role in CRC initiation and progression [47]. The correlation of HIF-1α and β-catenin is commonly detected in cancer and indicates malignant phenotypes [48]. We demonstrated that β-catenin is involved in the stabilization and translocation of HIF-1α from the cytoplasm to the nucleus in 5-FU resistance. The β-catenin transcriptional co-factor TCF1, but not TCF4, interacts with the HIF-1α/β-catenin complex. In-depth research on the mechanisms of the HIF-1α/β-catenin complex and co-factor TCFs can help us to identify more potential targets for molecular inhibitors. We found that high levels of HIF-1α in 5-FU resistance in CRC were attributed to increased stability, prolonged half-life, cellular translocation, and up-regulated transcription. ROS/PI3K/Akt signaling and Wnt/β-catenin signaling are responsible at least in part for the comprehensive changes of HIF-1α and the metabolic reprogramming in our 5-FU resistance models.

Data from CRC patients with preoperative fluorouracil analog-based chemotherapy revealed that increased HIF-1α expression in primary tumors or CTCs may suggest chemotherapy resistance, and high HIF-1α expression was associated with a significantly decreased disease-free survival in patients who had been previously treated with fluorouracil analog. Detection of CTCs in liquid biopsies is a promising strategy for diagnosing cancer, monitoring relapse and metastasis, and evaluating cancer prognosis and therapy [36, 37]. Studies showed that the presence of CTCs in CRC patients is a strong predictor of poor prognosis [37]. Our preliminary studies also suggested that CTCs have great potential to serve as a prognostic biomarker of CRC for evaluating the outcome of 5-FU-based chemotherapy, especially CTCs in the reflux veins of tumor, which carry more accurate information about the primary tumor. Analyses with larger samples in multi-center, prospective randomized and controlled trials are needed to draw more definitive conclusions.

## Conclusions

**Fig. 9:**
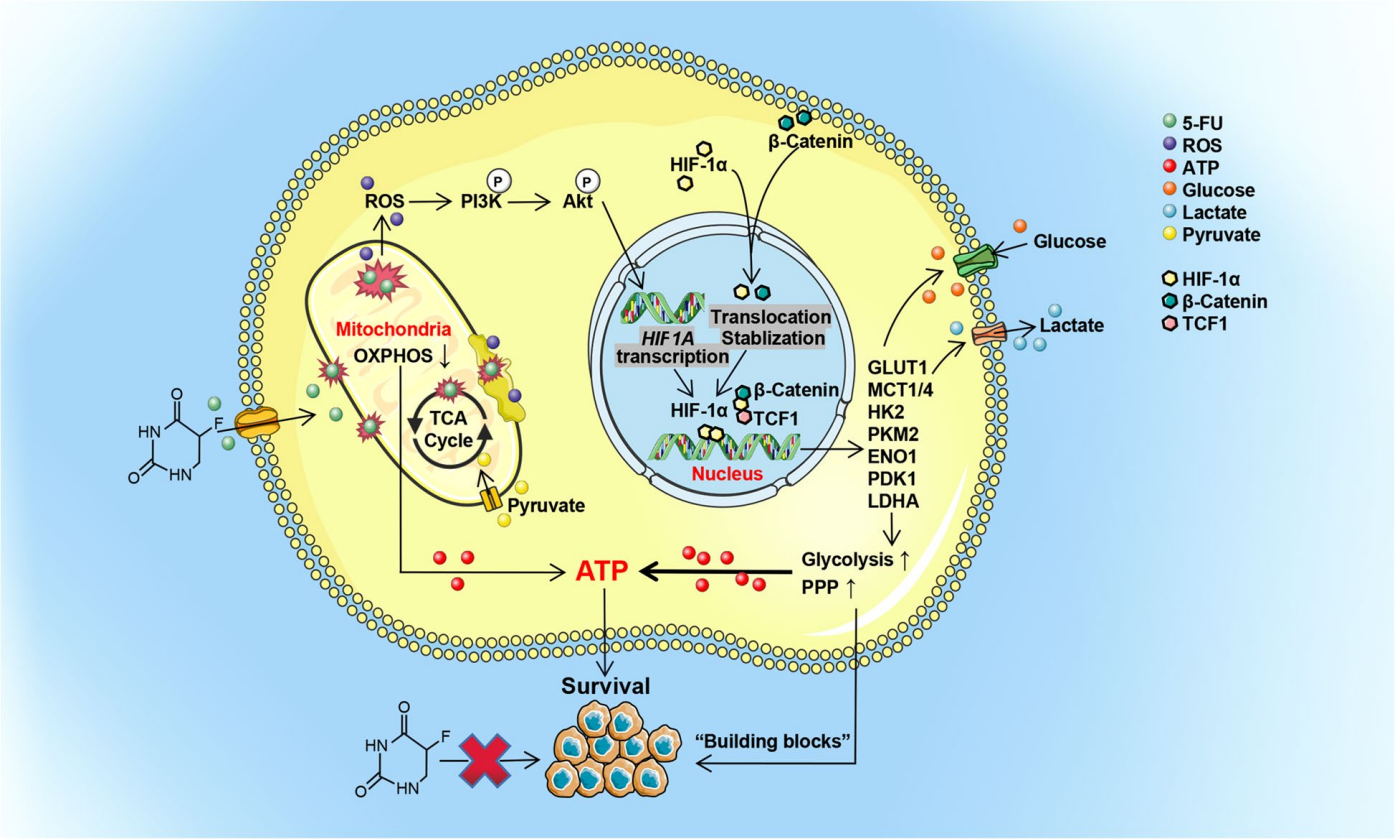
Schematic illustration of HIF-1α-induced glucose metabolic reprogramming imparts 5-FU resistance in CRC. The cumulative mitochondrial damage in CRC is the primary driver of glucose metabolic reprogramming, which is a shift from OXPHOS to glycolysis and PPP. The 5-FU resistance phenotype in CRC is derived from energy metabolism reprogramming, which arises from HIF-1α upregulation in non-classical ways, by ROS and the Wnt/β-catenin signaling pathway, independently from external oxygen concentrations

Numerous strategies based on molecular targets have been proposed to reverse 5-FU resistance in CRC and improve 5-FU treatment outcomes [3, 13, 57]; however, these efforts have not achieved success. Here, we present an overview of our results. The cumulative mitochondrial damage in CRC was the primary driver of glucose metabolic reprogramming, which is represented by high fluxes of glycolysis and PPP. The metabolic shift contributes considerable energy and biosynthetic substrates. Metabolic reprogramming confers additional tumor phenotypes, and our evidence indicates that the 5-FU resistance phenotype in CRC is derived from energy metabolism remodeling. Aim at the metabolic characteristics of 5-FU resistant CRC, we selected HIF-1α as a validated therapeutic target. Inhibition of HIF-1α contributes to overall suppression of glycolytic enzymes and glucose/lactate transporters, and no alternative pathway of energy supply is activated because of inherent mitochondria aberrations in 5-FU-R cells. We also identified oxygen-independent regulations of HIF-1α in 5-FU resistance, which may lead to identifying more therapeutic targets (Fig. 9). Additionally, it was initially demonstrated that HIF-1α may be a reliable biomarker for assessment, prognosis, and tracking of treatment response in CRC patients with 5-FU treatment.

## Supplementary Information

**Additional file 1: Figure S1.** Establishment and characterization of the acquired 5-FU-R CRC cell line models, related to Fig. 1. **a.** Acquired 5-FU-R CRC cell line models (HCT8, HCT15, and LoVo) were established by inducing WT CRC cells with a gradient increased 5-FU concentrations over a period of approximately 8 months. **b.** Phalloidin (green) and DAPI (blue) staining to visualize the cell morphology and nuclei. Scale bar = 50 μm. **c.** EdU incorporation assays of cell proliferation with or without 10^− 5^ M 5-FU treatment. EdU positive cells (red), nucleus (blue). Scale bar = 100 μm. **d.** Cell cycle distribution with or without 10^− 5^ M 5-FU treatment. **e.** Cell apoptosis with or without 10^− 5^ M 5-FU treatment. The four populations were distinguished as follows: viable cells (PE Annexin-V and 7-AAD negative), early apoptotic cells (PE Annexin-V positive and 7-AAD negative), late apoptotic cells (PE Annexin-V and 7-AAD positive), and dead cells (PE Annexin-V negative and 7-AAD positive). **f.** Comparing basal respiration, maximal respiration, and spare capacity in 5-FU-R CRC cells to WT CRC cells for assessing the mitochondrial respiration function. For all studies n was ≥3. Data are means ± SEM. Bar chart data were compared by Student’s t-test (* *p <* 0.05, ** *p <* 0.01, and *** *p <* 0.001).

**Additional file 2: Figure S2.** Increased glucose and lactate utilization fuels 5-FU resistance in 5-FU-R CRC cells, related to Fig. 2. **a.** RT-qPCR analysis for GLUTs genes in 5-FU-R CRC cells relative to WT CRC cells. *ACTB* was used as the internal reference. **b.** ^1^H-NMR intracellular metabolomics analysis for the difference of intracellular lactate in WT and 5-FU-R CRC cells. Chemical shift of lactate is 1.33(d), 4.12(q). **c.** RT-qPCR analysis for MCTs genes in 5-FU-R CRC cells relative to WT CRC cells. *ACTB* was used as the internal reference. **d.** Western blots of LDHA in WT and 5-FU-R cells. β-Actin was used as the internal reference. **e.** The enzyme activity of LDH was measured by colorimetric analysis. Enzyme activity was normalized to total protein concentration. f. Mitochondria stained by Mito-tracker (red), immunocytofluorescence staining of LDHA (green), and nuclear stained by DAPI (blue). There is mitochondria/LDHA co-localization displayed by the intense yellow color in the merged image. Scale bar = 10 μm. For all studies *n* ≥ 3. Data are presented as means ± SEM. Bar chart data were compared by Student’s t-test (** *p <* 0.01, and *** *p <* 0.001).

**Additional file 3: Figure S3.** Increased glycolysis and PPP in 5-FU-R CRC cells, related to Fig. 3. **a.** Unsupervised hierarchical clustering of differential glucose metabolite pools in WT and 5-FU-R CRC cells. **b.** Comparing basal glycolysis, glycolytic capacity and glycolytic reserve in 5-FU-R CRC cells to WT CRC cells for assessing the glycolytic stress. For all studies n ≥ 3. Data are presented as means ± SEM. Bar chart data were compared by Student’s-t test (* *p <* 0.05, ** *p <* 0.01, and *** *p <* 0.001).

**Additional file 4: Figure S4.** HIF-1α is a prognostic biomarker and regulator of 5-FU resistance in CRC, related to Figs. 4 and 5. **a.** *HIF1A* mRNA expressions were obtained and compared from 2 GEO datasets. **b.** Nucleus and cytoplasm distribution of HIF-1α was analyzed by Western blots. Lamin B1 is a nuclear protein reference, and β-Actin is a cytoplasmic marker. **c.** Expression of HIF-1a in WT CRC cells versus 5-FU-R CRC cells. Cells were cultured under normoxia (20% oxygen) or hypoxia (1% oxygen). β-Actin was used as an internal reference. **d.** WT and 5-FU-R CRC cells were stably knocked down for *HIF1A* using shRNA. HIF-1a was confirmed by Western blotting, using β-Actin as a loading control. **e.** Western blots of HIF-1α in 11 CRC cell lines (HCT8, DLD-1, HCT116, HT29, SW480, SW1116, DiFi, Caco-2, HCT15, T84, and LoVo). β-Actin was used as the internal reference. **f.** CCK8 assays to assess 5-FU sensitivity of 11 CRC cell lines, and cells were treated with an increasing concentrations of 5-FU for 72 h. All experiments were performed with 6 replicates. Data are presented as means ± SEM. Bar chart data were compared by Student’s t-test (ns = not significant).

**Additional file 5: Figure S5.** Both *HIF1A* knock-down and pharmacological inhibition of HIF-1α are effective for reducing 5-FU resistance in vivo, related to Fig. 6. **a.** Representative images of IHC staining of HIF-1α, GLUT1, HK2, PKM2, LDHA, and MCT4 on tumor sections. Scale bar = 100 μm. **b.** Effect of *HIF1A* knockout on 5-FU resistance in subcutaneously-implanted WT or 5-FU-R cells in a nude mouse model. One week after subcutaneous injection, the mice were treated intraperitoneally with 25 mg/kg 5-FU or saline three times a week. Body weights of mice with the indicated treatments (b). **c-d.** Effect of IDF-11774 on 5-FU resistance in subcutaneously-implanted WT or 5-FU-R cells in a nude mouse model. Tumors harvested from subcutaneously-implanted nude mice treated with saline (control), 5-FU alone (25 mg/kg, three times a week), IDF-11774 alone (30 mg/kg, twice a week) or 5-FU together with IDF-11774. Body weights of mice with the indicated treatments (c). Representative images of IHC staining for Ki-67, scale bar = 100 μm (d). **e-f.** Effect of IDF-11774 on 5-FU resistance in a PDXs NOD/scid mouse model. Tumors harvested from subcutaneously-implanted nude mice treated with saline (control), 5-FU alone (25 mg/kg, three times a week), IDF-11774 alone (30 mg/kg, twice a week) or 5-FU together with IDF-11774. Body weights of mice with the indicated treatments (e). Representative images of IHC staining for Ki-67, scale bar = 100 μm (f).

**Additional file 6: Figure S6.** ROS/PI3K/AKT pathway activation boosts HIF-1α levels and induces 5-FU resistance, related to Fig. 7. **a.** RT-qPCR analysis for ROS scavenging enzymes genes in 5-FU-R CRC cells relative to WT CRC cells. *ACTB* was used as the internal reference. **b.** The activities of CAT, GPx, and SOD enzymes of 5-FU-R CRC cells relative to WT CRC cells as determined by colorimetric analysis. Enzyme activity was normalized to total protein concentration. **c.** Representative IHC staining images of p-Akt and HIF-1α in CRC patients received preoperative fluorouracil analog-based chemotherapy. Scale bar = 100 μm. **d.** 5-FU-R cells were treated with 25 μM LY294002 for 48 h. RT-qPCR analysis for the gene expressions of *HIF1A*. *ACTB* is used as an internal reference. **e.** Effect of *HIF1A* knock-down on PI3K/AKT pathway. β-Actin was used as an internal reference. For all studies *n* ≥ 3. Data are presented as means ± SEM. Data were analyzed by Student’s t-test or ANOVA (ns = not significant, * *p <* 0.05, ** *p <* 0.01, and *** *p <* 0.001).

**Additional file 7: Figure S7.** The HIF-1α/β-catenin transcriptional complex contributes to 5-FU resistance and glycolytic activity, related to Fig. 8. **a.** WT and 5-FU-R cells cultured with 100 μM CHX for 1 h, 2 h, and 6 h to compare the stability of β-catenin. β-Actin was used as the internal reference. **b.** Anti-β-catenin co-immunoprecipitation experiments to identify interactions between β-catenin, HIF-1α, TCF1, and TCF4 in WT and 5-FU-R HCT15 and LoVo cells. **c.** Anti-HIF-1α co-immunoprecipitation experiments to identify interactions between HIF-1α, β-catenin, TCF1, and TCF4 in WT and 5-FU-R HCT15 and LoVo cells. Representative IHC staining images of HIF-1α, β-catenin, and TCF1 in CRC patients received preoperative fluorouracil analog-based chemotherapy. Scale bar = 100 μm.

**Additional file 8: Table S1.** Clinicopathological data. Clinicopathological data of CRC patients were gathered from the medical records and pathologic data.

**Additional file 9: Table S2.** Reagent used in this study.

**Additional file 10: Table S3.** Primers used in this study.

## Data Availability

The authors declare that all data and materials supporting the findings of this study are available in this article and its supplementary files.

## Abbreviations

5-FU: 5-fluorouracil
CRC: Colorectal cancer
HIF-1α: Hypoxia inducible factor 1α
OXPHOS: Oxidative phosphorylation
PPP: Pentose phosphate pathway
ROS: Reactive oxygen species
GEO: Gene Expression Omnibus
SiRNA: Small interfering RNA
ShRNA: Short hairpin RNA
PFA: Paraformaldehyde
UHPLC-MS/MS: Ultra-high pressure liquid chromatography-coupled tandem mass spectrometry
OCR: Oxygen consumption rate
ECAR: Extracellular acidification rate
Co-IP: Co-immunoprecipitation
RT-qPCR: Real-time quantitative PCR
CDX: Cell-derived xenograft
PDX: Patient derived xenograft
MRS: Magnetic resonance spectroscopy
CTC: Circulating tumor cell
IHC: Immunohistochemistry
LDH: Lactate dehydrogenase
CAT: Catalase
GPx: Glutathione peroxidase
SOD: Superoxide dismutase
GLUT: Glucose transporters
MCT: Monocarboxylic acid transporters
