# Supplementary material for: ROS/PI3K/Akt and Wnt/β-catenin signalings activate HIF-1α-induced metabolic reprogramming to impart 5-fluorouracil resistance in colorectal cancer
Source: J Exp Clin Cancer Res. 2022 Jan 8;41:15. doi: 10.1186/s13046-021-02229-6 (PMC8742403; doi:10.1186/s13046-021-02229-6)
Supplement: Supplementary file 1 — Additional file 1: Figure S1. Establishment and characterization of the acquired 5-FU-R CRC cell line models, related to Fig. 1. a. Acquired 5-FU-R CRC cell line models (HCT8, HCT15, and LoVo) were established by inducing WT CRC cells with a gradient increased 5-FU concentrations over a period of approximately 8 months. b. Phalloidin (green) and DAPI (blue) staining to visualize the cell morphology and nuclei. Scale bar = 50 μm. c. EdU incorporation assays of cell proliferation with or without 10− 5 M 5-FU treatment. EdU positive cells (red), nucleus (blue). Scale bar = 100 μm. d. Cell cycle distribution with or without 10− 5 M 5-FU treatment. e. Cell apoptosis with or without 10− 5 M 5-FU treatment. The four populations were distinguished as follows: viable cells (PE Annexin-V and 7-AAD negative), early apoptotic cells (PE Annexin-V positive and 7-AAD negative), late apoptotic cells (PE Annexin-V and 7-AAD positive), and dead cells (PE Annexin-V negative and 7-AAD positive). f. Comparing basal respiration, maximal respiration, and spare capacity in 5-FU-R CRC cells to WT CRC cells for assessing the mitochondrial respiration function. For all studies n was ≥3. Data are means ± SEM. Bar chart data were compared by Student’s t-test (* p < 0.05, ** p < 0.01, and *** p < 0.001). [file 13046_2021_2229_MOESM1_ESM.pdf]

Supplementary Information

ROS/PI3K/Akt and Wnt/β-catenin signalings activate HIF-1α-induced metabolic reprogramming to impart 5-fluorouracil resistance in colorectal cancer

Authors

Shuohui Dong[1], Shuo Liang[2], Zhiqiang Cheng[3], Xiang Zhang[3], Li Luo[4], Linchuan Li[1], Wenjie Zhang[1], Songhan Li[1], Qian Xv[1], Mingwei Zhong[1], Jiankang Zhu[1], Guangyong Zhang[1], Sanyuan Hu[1,*]

[1] Department of General Surgery, Shandong Qianfoshan Hospital, Cheeloo College of Medicine, Shandong University, Jinan, Shandong Province 250014, China.

[2] Department of Otolaryngology-Head and Neck Surgery, Shandong Provincial ENT Hospital, Cheeloo College of Medicine, Shandong University, Jinan, Shandong Province 250023, China.

[3]Department of General Surgery, Qilu Hospital, Cheeloo College of Medicine, Shandong University, Jinan, Shandong Province 250012, China.

[4] Department of Cardiac Surgery, the First Affiliated Hospital, Sun Yat-sen University, Guangzhou , Guangdong Province 510080, China.

[*]Correspondence: Sanyuan Hu (email: drsanyuanhu@163.com)

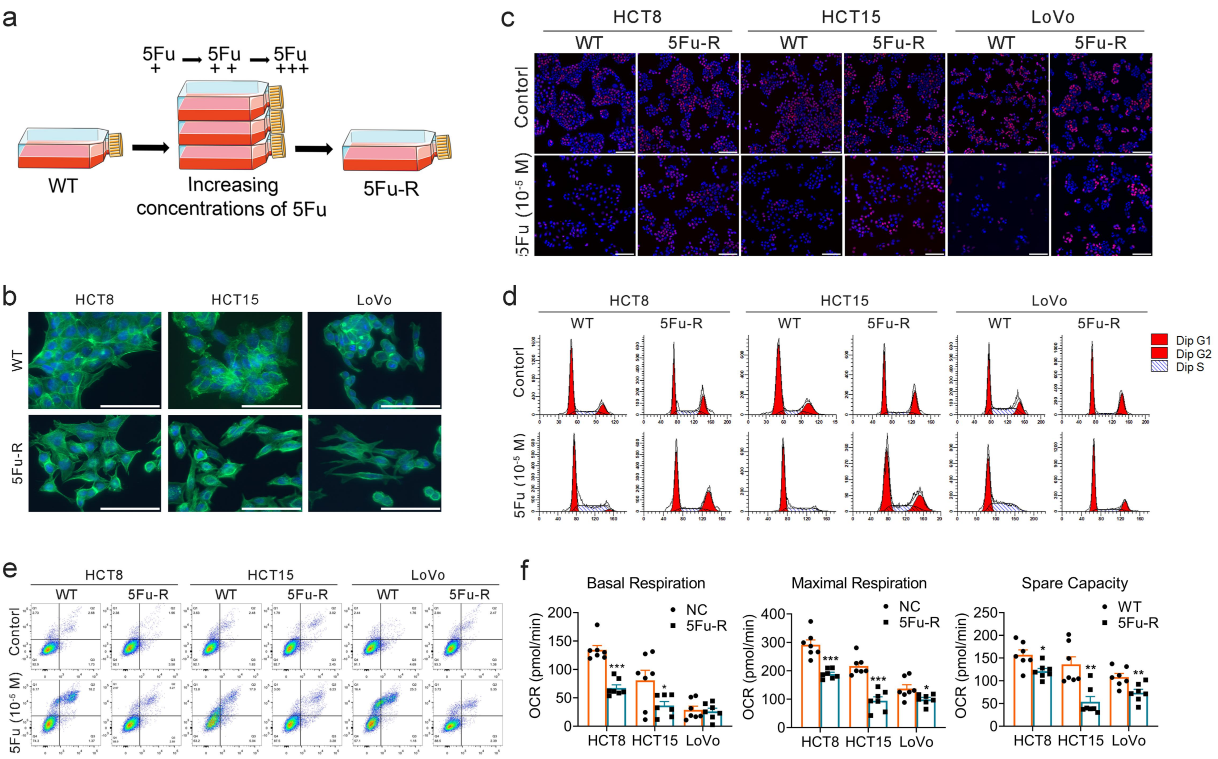

Additional file 1: Fig. S1. stablishment and characterization of the acquired 5-FU-R CRC cell line models, related to Fig. 1.

a. Acquired 5-FU-R CRC cell line models (HCT8, HCT15, and LoVo) were established by inducing WT CRC cells with a gradient increased 5-FU concentrations over a period of approximately 8 months.

b. Phalloidin (green) and DAPI (blue) staining to visualize the cell morphology and nuclei. Scale bar = 50 μm.

c. EdU incorporation assays of cell proliferation with or without 10^{-5}M 5-FU treatment. EdU positive cells (red), nucleus (blue). Scale bar = 100 μm.

d. Cell cycle distribution with or without 10^{-5}M 5-FU treatment.

e. Cell apoptosis with or without 10^{-5}M 5-FU treatment. The four populations were distinguished as follows: viable cells (PE Annexin-V and 7-AAD negative), early

apoptotic cells (PE Annexin-V positive and 7-AAD negative), late apoptotic cells (PE Annexin-V and 7-AAD positive), and dead cells (PE Annexin-V negative and 7-AAD positive).

f. Comparing basal respiration, maximal respiration, and spare capacity in 5-FU-R CRC cells to WT CRC cells for assessing the mitochondrial respiration function.

For all studies n was ≥3. Data are means ± SEM. Bar chart data were compared by Student's t-test (* $p < 0.05$, ** $p < 0.01$, and *** $p < 0.001$).
