# Supplementary material for: ROS/PI3K/Akt and Wnt/β-catenin signalings activate HIF-1α-induced metabolic reprogramming to impart 5-fluorouracil resistance in colorectal cancer
Source: J Exp Clin Cancer Res. 2022 Jan 8;41:15. doi: 10.1186/s13046-021-02229-6 (PMC8742403; doi:10.1186/s13046-021-02229-6)
Supplement: Supplementary file 2 — Additional file 2: Figure S2. Increased glucose and lactate utilization fuels 5-FU resistance in 5-FU-R CRC cells, related to Fig. 2. a. RT-qPCR analysis for GLUTs genes in 5-FU-R CRC cells relative to WT CRC cells. ACTB was used as the internal reference. b. 1H-NMR intracellular metabolomics analysis for the difference of intracellular lactate in WT and 5-FU-R CRC cells. Chemical shift of lactate is 1.33(d), 4.12(q). c. RT-qPCR analysis for MCTs genes in 5-FU-R CRC cells relative to WT CRC cells. ACTB was used as the internal reference. d. Western blots of LDHA in WT and 5-FU-R cells. β-Actin was used as the internal reference. e. The enzyme activity of LDH was measured by colorimetric analysis. Enzyme activity was normalized to total protein concentration. f. Mitochondria stained by Mito-tracker (red), immunocytofluorescence staining of LDHA (green), and nuclear stained by DAPI (blue). There is mitochondria/LDHA co-localization displayed by the intense yellow color in the merged image. Scale bar = 10 μm. For all studies n ≥ 3. Data are presented as means ± SEM. Bar chart data were compared by Student’s t-test (** p < 0.01, and *** p < 0.001). [file 13046_2021_2229_MOESM2_ESM.pdf]

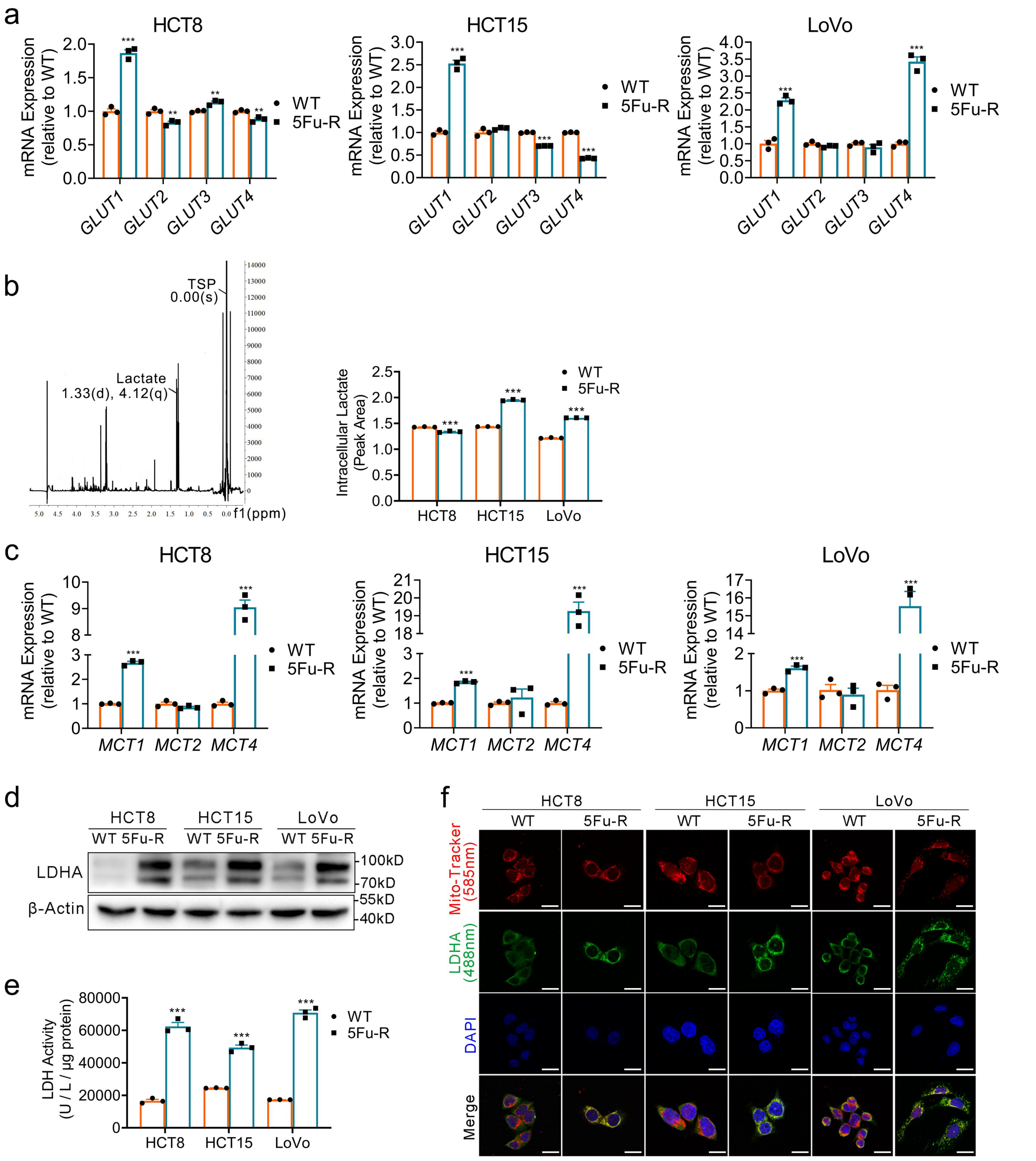

Additional file 2: Fig. S2. ncreased glucose and lactate utilization fuels 5-FU resistance in 5-FU-R CRC cells, related to Fig. 2.

a. RT-qPCR analysis for GLUTs genes in 5-FU-R CRC cells relative to WT CRC cells. *ACTB* was used as the internal reference.

b. ^{1}H-NMR intracellular metabolomics analysis for the difference of intracellular lactate in WT and 5-FU-R CRC cells. Chemical shift of lactate is 1.33(d), 4.12(q).

c. RT-qPCR analysis for MCTs genes in 5-FU-R CRC cells relative to WT CRC cells.

ACTB was used as the internal reference.

d. Western blots of LDHA in WT and 5-FU-R cells. β-Actin was used as the internal reference.

e. The enzyme activity of LDH was measured by colorimetric analysis. Enzyme activity was normalized to total protein concentration.

f. Mitochondria stained by Mito-tracker (red), immunocytofluorescence staining of LDHA (green), and nuclear stained by DAPI (blue). There is mitochondria/LDHA co-localization displayed by the intense yellow color in the merged image. Scale bar = 10 μm.

For all studies n ≥3. Data are presented as means ± SEM. Bar chart data were compared by Student's t-test (** $p < 0.01$, and *** $p < 0.001$).
