# Supplementary material for: ROS/PI3K/Akt and Wnt/β-catenin signalings activate HIF-1α-induced metabolic reprogramming to impart 5-fluorouracil resistance in colorectal cancer
Source: J Exp Clin Cancer Res. 2022 Jan 8;41:15. doi: 10.1186/s13046-021-02229-6 (PMC8742403; doi:10.1186/s13046-021-02229-6)
Supplement: Supplementary file 3 — Additional file 3: Figure S3. Increased glycolysis and PPP in 5-FU-R CRC cells, related to Fig. 3. a. Unsupervised hierarchical clustering of differential glucose metabolite pools in WT and 5-FU-R CRC cells. b. Comparing basal glycolysis, glycolytic capacity and glycolytic reserve in 5-FU-R CRC cells to WT CRC cells for assessing the glycolytic stress. For all studies n ≥ 3. Data are presented as means ± SEM. Bar chart data were compared by Student’s-t test (* p < 0.05, ** p < 0.01, and *** p < 0.001). [file 13046_2021_2229_MOESM3_ESM.pdf]

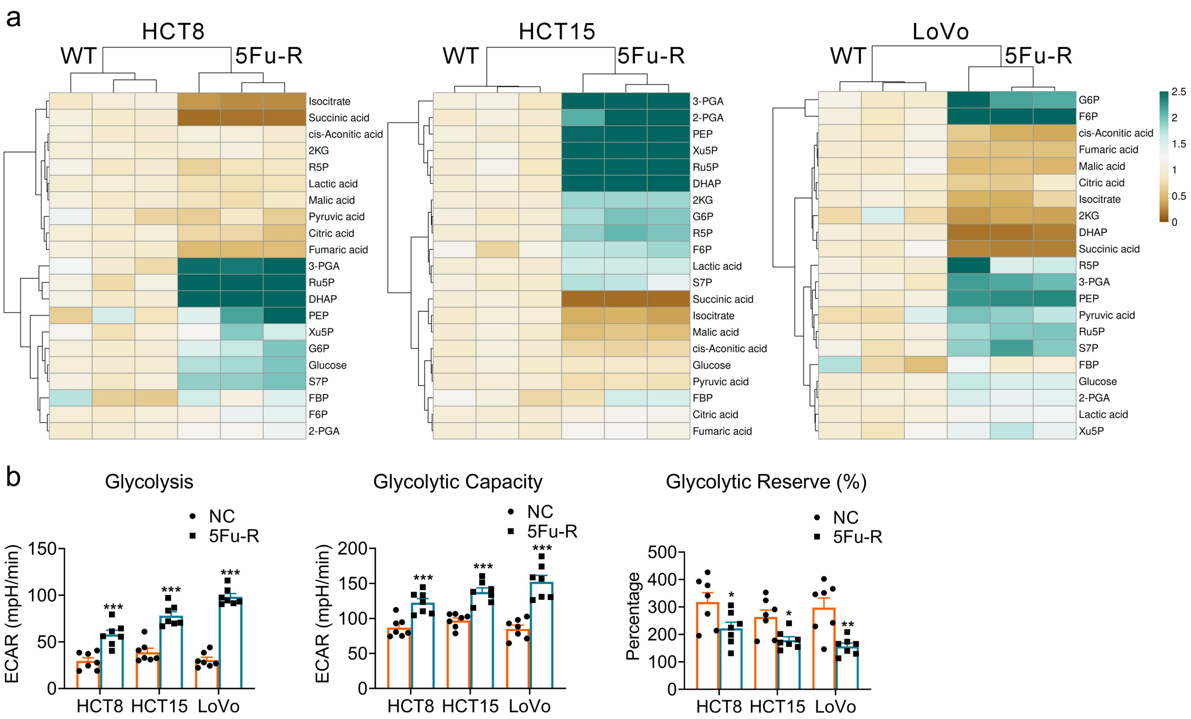

Additional file 3: Fig. S3. Increased glycolysis and PPP in 5-FU-R CRC cells, related to Fig. 3.

a. Unsupervised hierarchical clustering of differential glucose metabolite pools in WT and 5-FU-R CRC cells.

b. Comparing basal glycolysis, glycolytic capacity and glycolytic reserve in 5-FU-R CRC cells to WT CRC cells for assessing the glycolytic stress.

For all studies n ≥3. Data are presented as means ± SEM. Bar chart data were compared by Student's-t test (* $p < 0.05$, ** $p < 0.01$, and *** $p < 0.001$).
