# Supplementary material for: ROS/PI3K/Akt and Wnt/β-catenin signalings activate HIF-1α-induced metabolic reprogramming to impart 5-fluorouracil resistance in colorectal cancer
Source: J Exp Clin Cancer Res. 2022 Jan 8;41:15. doi: 10.1186/s13046-021-02229-6 (PMC8742403; doi:10.1186/s13046-021-02229-6)
Supplement: Supplementary file 4 — Additional file 4: Figure S4. HIF-1α is a prognostic biomarker and regulator of 5-FU resistance in CRC, related to Figs. 4 and 5. a. HIF1A mRNA expressions were obtained and compared from 2 GEO datasets. b. Nucleus and cytoplasm distribution of HIF-1α was analyzed by Western blots. Lamin B1 is a nuclear protein reference, and β-Actin is a cytoplasmic marker. c. Expression of HIF-1a in WT CRC cells versus 5-FU-R CRC cells. Cells were cultured under normoxia (20% oxygen) or hypoxia (1% oxygen). β-Actin was used as an internal reference. d. WT and 5-FU-R CRC cells were stably knocked down for HIF1A using shRNA. HIF-1a was confirmed by Western blotting, using β-Actin as a loading control. e. Western blots of HIF-1α in 11 CRC cell lines (HCT8, DLD-1, HCT116, HT29, SW480, SW1116, DiFi, Caco-2, HCT15, T84, and LoVo). β-Actin was used as the internal reference. f. CCK8 assays to assess 5-FU sensitivity of 11 CRC cell lines, and cells were treated with an increasing concentrations of 5-FU for 72 h. All experiments were performed with 6 replicates. Data are presented as means ± SEM. Bar chart data were compared by Student’s t-test (ns = not significant). [file 13046_2021_2229_MOESM4_ESM.pdf]

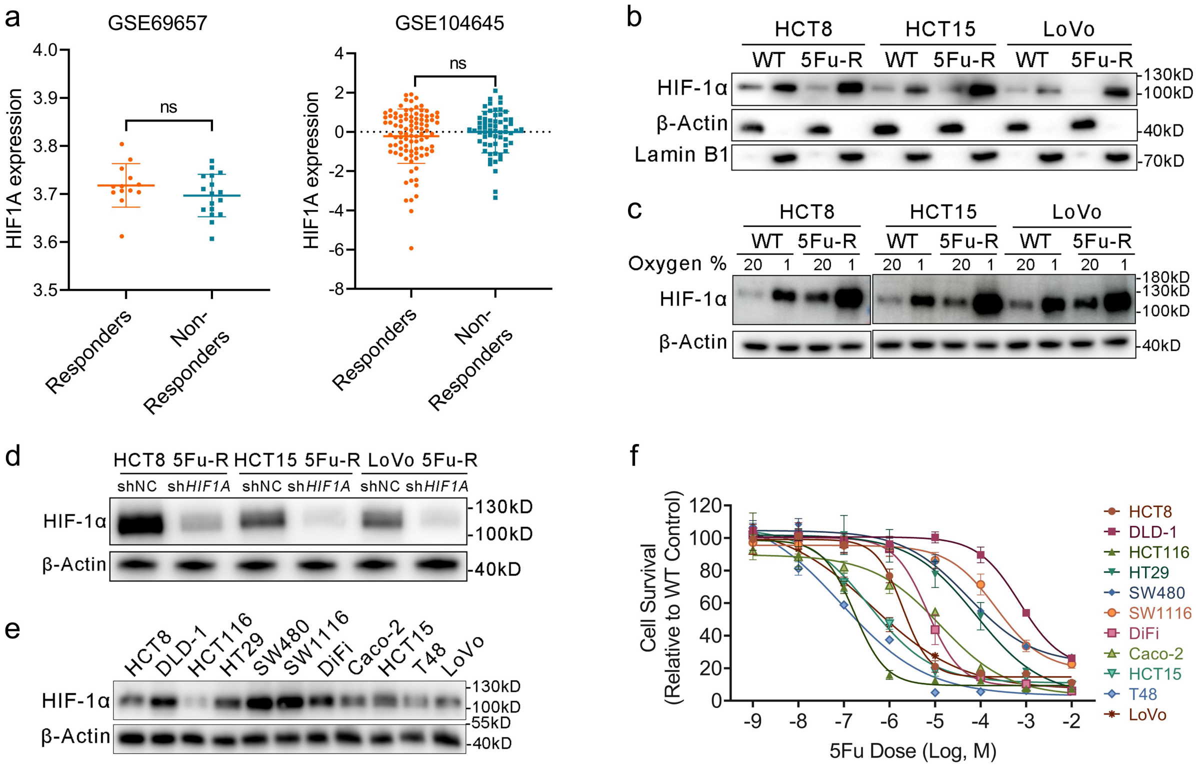

Additional file 4: Fig. S4. HIF-1α is a prognostic biomarker and regulator of 5-FU resistance in CRC, related to Fig. 4 and Fig. 5.

a. *HIF1A* mRNA expressions were obtained and compared from 2 GEO datasets.

b. Nucleus and cytoplasm distribution of HIF-1α was analyzed by Western blots. Lamin B1 is a nuclear protein reference, and β-Actin is a cytoplasmic marker.

c. Expression of HIF-1a in WT CRC cells versus 5-FU-R CRC cells. Cells were cultured under normoxia (20% oxygen) or hypoxia (1% oxygen). β-Actin was used as an internal reference.

d. WT and 5-FU-R CRC cells were stably knocked down for *HIF1A* using shRNA. HIF-1a was confirmed by Western blotting, using β-Actin as a loading control.

e. Western blots of HIF-1α in 11 CRC cell lines (HCT8, DLD-1, HCT116, HT29, SW480, SW1116, DiFi, Caco-2, HCT15, T84, and LoVo). β-Actin was used as the

internal reference.

f. CCK8 assays to assess 5-FU sensitivity of 11 CRC cell lines, and cells were treated with an increasing concentrations of 5-FU for 72 hours. All experiments were performed with 6 replicates.

Data are presented as means ± SEM. Bar chart data were compared by Student's t-test (ns = not significant).
