# Supplementary material for: ROS/PI3K/Akt and Wnt/β-catenin signalings activate HIF-1α-induced metabolic reprogramming to impart 5-fluorouracil resistance in colorectal cancer
Source: J Exp Clin Cancer Res. 2022 Jan 8;41:15. doi: 10.1186/s13046-021-02229-6 (PMC8742403; doi:10.1186/s13046-021-02229-6)
Supplement: Supplementary file 5 — Additional file 5: Figure S5. Both HIF1A knock-down and pharmacological inhibition of HIF-1α are effective for reducing 5-FU resistance in vivo, related to Fig. 6. a. Representative images of IHC staining of HIF-1α, GLUT1, HK2, PKM2, LDHA, and MCT4 on tumor sections. Scale bar = 100 μm. b. Effect of HIF1A knockout on 5-FU resistance in subcutaneously-implanted WT or 5-FU-R cells in a nude mouse model. One week after subcutaneous injection, the mice were treated intraperitoneally with 25 mg/kg 5-FU or saline three times a week. Body weights of mice with the indicated treatments (b). c-d. Effect of IDF-11774 on 5-FU resistance in subcutaneously-implanted WT or 5-FU-R cells in a nude mouse model. Tumors harvested from subcutaneously-implanted nude mice treated with saline (control), 5-FU alone (25 mg/kg, three times a week), IDF-11774 alone (30 mg/kg, twice a week) or 5-FU together with IDF-11774. Body weights of mice with the indicated treatments (c). Representative images of IHC staining for Ki-67, scale bar = 100 μm (d). e-f. Effect of IDF-11774 on 5-FU resistance in a PDXs NOD/scid mouse model. Tumors harvested from subcutaneously-implanted nude mice treated with saline (control), 5-FU alone (25 mg/kg, three times a week), IDF-11774 alone (30 mg/kg, twice a week) or 5-FU together with IDF-11774. Body weights of mice with the indicated treatments (e). Representative images of IHC staining for Ki-67, scale bar = 100 μm (f). [file 13046_2021_2229_MOESM5_ESM.pdf]

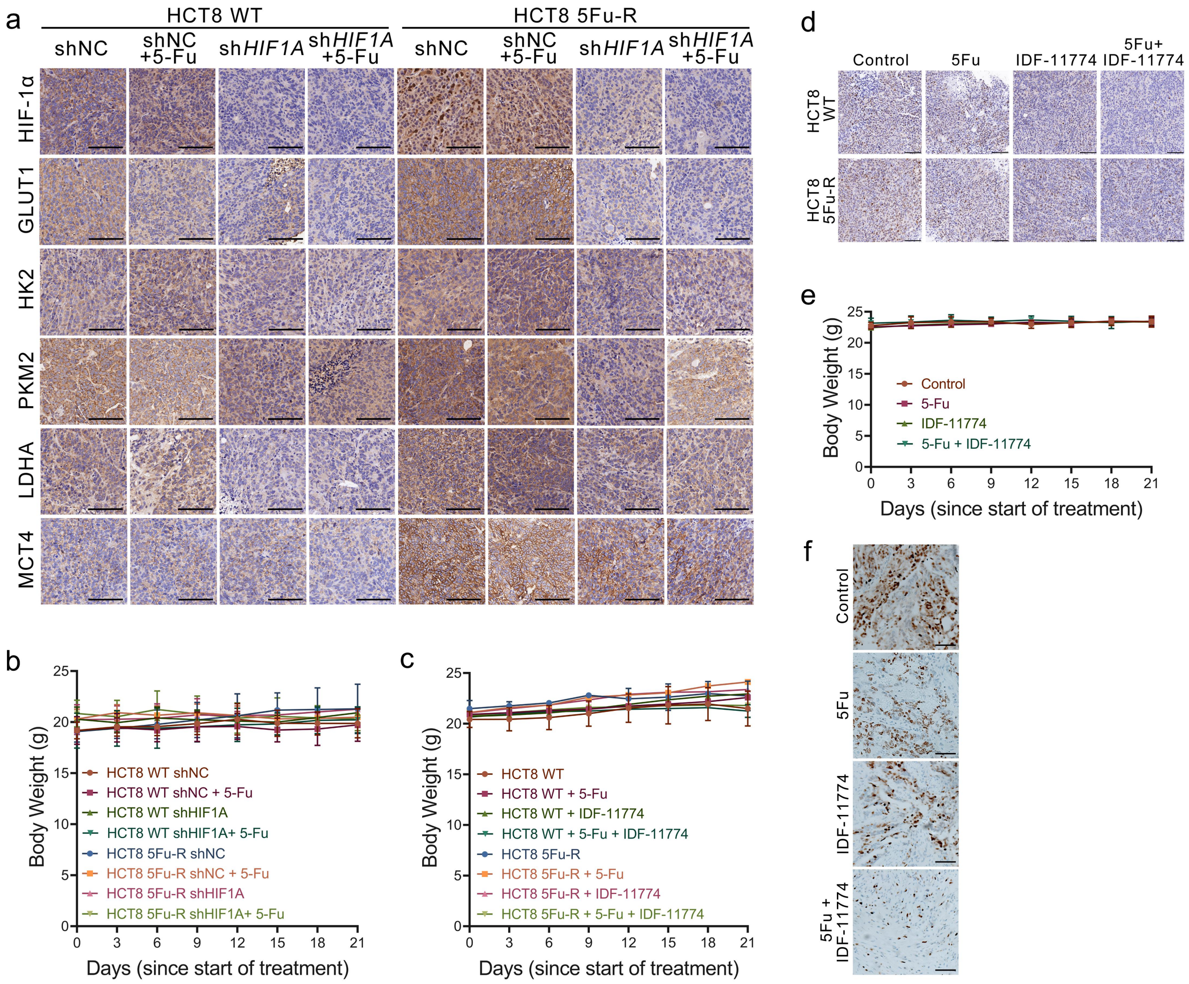

Additional file 5: Fig. S5. Both *HIF1A* knock-down and pharmacological inhibition of HIF-1α are effective for reducing 5-FU resistance in vivo, related to Fig. 6.

a. Representative images of IHC staining of HIF-1α, GLUT1, HK2, PKM2, LDHA, and MCT4 on tumor sections. Scale bar = 100μm.

b. Effect of *HIF1A* knockout on 5-FU resistance in subcutaneously-implanted WT or 5-FU-R cells in a nude mouse model. One week after subcutaneous injection, the mice were treated intraperitoneally with 25 mg/kg 5-FU or saline three times a week. Body weights of mice with the indicated treatments (b).

c-d. Effect of IDF-11774 on 5-FU resistance in subcutaneously-implanted WT or

5-FU-R cells in a nude mouse model. Tumors harvested from subcutaneously-implanted nude mice treated with saline (control), 5-FU alone (25 mg/kg, three times a week), IDF-11774 alone (30 mg/kg, twice a week) or 5-FU together with IDF-11774. Body weights of mice with the indicated treatments (c). Representative images of IHC staining for Ki-67, scale bar = 100μm (d).

e-f. Effect of IDF-11774 on 5-FU resistance in a PDXs NOD/scid mouse model. Tumors harvested from subcutaneously-implanted nude mice treated with saline (control), 5-FU alone (25 mg/kg, three times a week), IDF-11774 alone (30 mg/kg, twice a week) or 5-FU together with IDF-11774. Body weights of mice with the indicated treatments (e). Representative images of IHC staining for Ki-67, scale bar = 100μm (f).
