# Supplementary material for: ROS/PI3K/Akt and Wnt/β-catenin signalings activate HIF-1α-induced metabolic reprogramming to impart 5-fluorouracil resistance in colorectal cancer
Source: J Exp Clin Cancer Res. 2022 Jan 8;41:15. doi: 10.1186/s13046-021-02229-6 (PMC8742403; doi:10.1186/s13046-021-02229-6)
Supplement: Supplementary file 6 — Additional file 6: Figure S6. ROS/PI3K/AKT pathway activation boosts HIF-1α levels and induces 5-FU resistance, related to Fig. 7. a. RT-qPCR analysis for ROS scavenging enzymes genes in 5-FU-R CRC cells relative to WT CRC cells. ACTB was used as the internal reference. b. The activities of CAT, GPx, and SOD enzymes of 5-FU-R CRC cells relative to WT CRC cells as determined by colorimetric analysis. Enzyme activity was normalized to total protein concentration. c. Representative IHC staining images of p-Akt and HIF-1α in CRC patients received preoperative fluorouracil analog-based chemotherapy. Scale bar = 100 μm. d. 5-FU-R cells were treated with 25 μM LY294002 for 48 h. RT-qPCR analysis for the gene expressions of HIF1A. ACTB is used as an internal reference. e. Effect of HIF1A knock-down on PI3K/AKT pathway. β-Actin was used as an internal reference. For all studies n ≥ 3. Data are presented as means ± SEM. Data were analyzed by Student’s t-test or ANOVA (ns = not significant, * p < 0.05, ** p < 0.01, and *** p < 0.001). [file 13046_2021_2229_MOESM6_ESM.pdf]

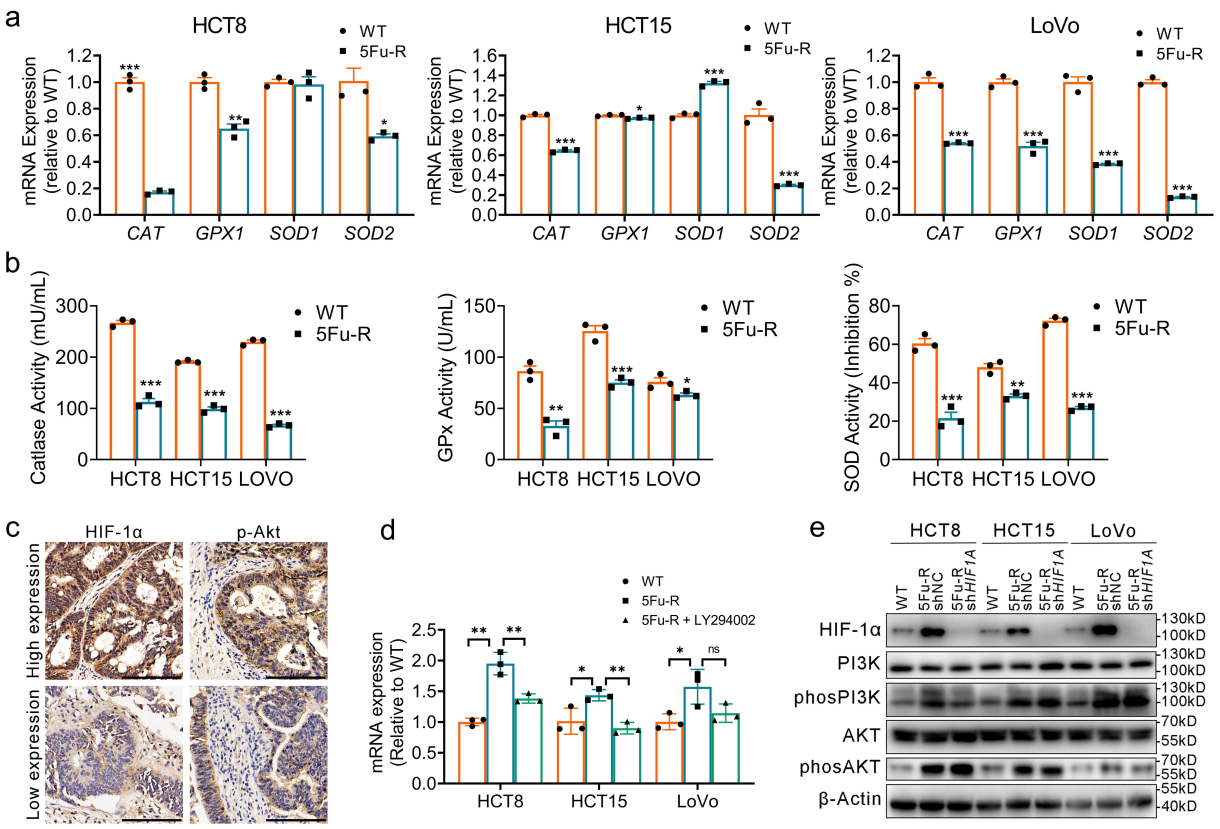

Additional file 6: Fig. S6. ROS/PI3K/AKT pathway activation boosts HIF-1α levels and induces 5-FU resistance, related to Fig. 7.

a. RT-qPCR analysis for ROS scavenging enzymes genes in 5-FU-R CRC cells relative to WT CRC cells. *ACTB* was used as the internal reference.

b. The activities of CAT, GPx, and SOD enzymes of 5-FU-R CRC cells relative to WT CRC cells as determined by colorimetric analysis. Enzyme activity was normalized to total protein concentration.

c. Representative IHC staining images of p-Akt and HIF-1α in CRC patients received preoperative fluorouracil analog-based chemotherapy. Scale bar = 100 μm.

d. 5-FU-R cells were treated with 25 μM LY294002 for 48 hours. RT-qPCR analysis for the gene expressions of *HIF1A*. *ACTB* is used as an internal reference.

e. Effect of *HIF1A* knock-down on PI3K/AKT pathway. β-Actin was used as an

internal reference.

For all studies n ≥3. Data are presented as means ± SEM. Data were analyzed by Student's t-test or ANOVA (ns = not significant, * $p < 0.05$, ** $p < 0.01$, and *** $p < 0.001$).
