# Supplementary material for: ROS/PI3K/Akt and Wnt/β-catenin signalings activate HIF-1α-induced metabolic reprogramming to impart 5-fluorouracil resistance in colorectal cancer
Source: J Exp Clin Cancer Res. 2022 Jan 8;41:15. doi: 10.1186/s13046-021-02229-6 (PMC8742403; doi:10.1186/s13046-021-02229-6)
Supplement: Supplementary file 7 — Additional file 7: Figure S7. The HIF-1α/β-catenin transcriptional complex contributes to 5-FU resistance and glycolytic activity, related to Fig. 8. a. WT and 5-FU-R cells cultured with 100 μM CHX for 1 h, 2 h, and 6 h to compare the stability of β-catenin. β-Actin was used as the internal reference. b. Anti-β-catenin co-immunoprecipitation experiments to identify interactions between β-catenin, HIF-1α, TCF1, and TCF4 in WT and 5-FU-R HCT15 and LoVo cells. c. Anti-HIF-1α co-immunoprecipitation experiments to identify interactions between HIF-1α, β-catenin, TCF1, and TCF4 in WT and 5-FU-R HCT15 and LoVo cells. Representative IHC staining images of HIF-1α, β-catenin, and TCF1 in CRC patients received preoperative fluorouracil analog-based chemotherapy. Scale bar = 100 μm. [file 13046_2021_2229_MOESM7_ESM.pdf]

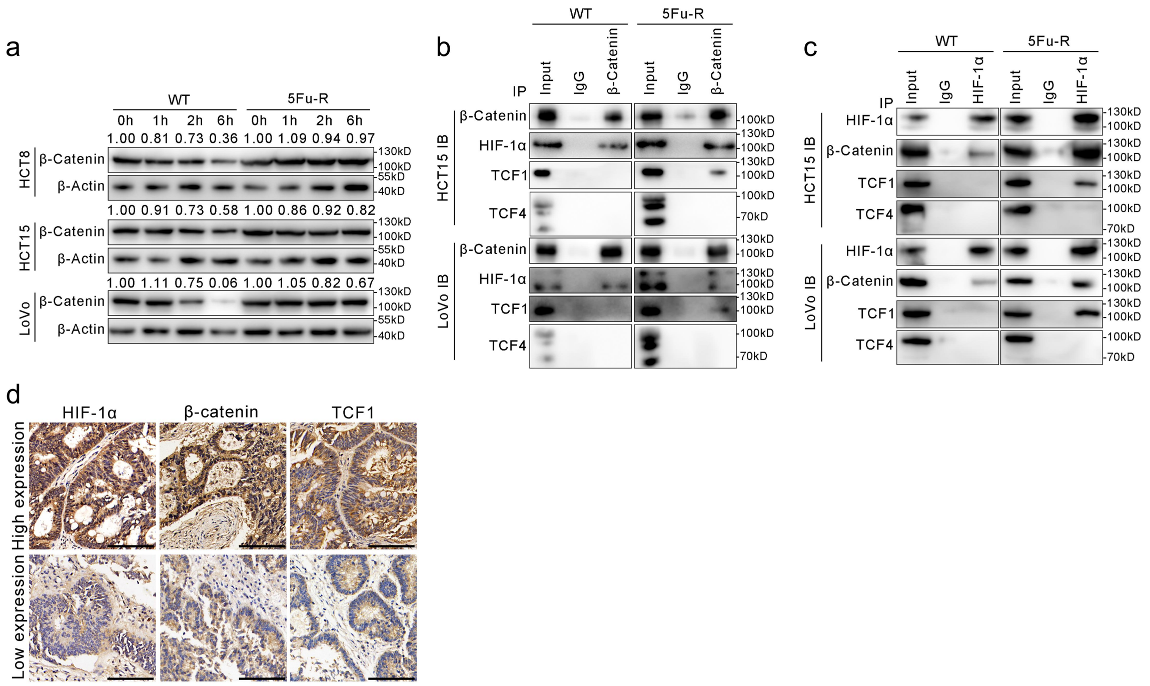

Additional file 7: Fig. S7. The HIF-1α/β-catenin transcriptional complex contributes to 5-FU resistance and glycolytic activity, related to Fig. 8.

a. WT and 5-FU-R cells cultured with 100 μM CHX for 1 hour, 2 hours, and 6 hours to compare the stability of β-catenin. β-Actin was used as the internal reference.

b. Anti-β-catenin co-immunoprecipitation experiments to identify interactions between β-catenin, HIF-1α, TCF1, and TCF4 in WT and 5-FU-R HCT15 and LoVo cells.

c. Anti-HIF-1α co-immunoprecipitation experiments to identify interactions between HIF-1α, β-catenin, TCF1, and TCF4 in WT and 5-FU-R HCT15 and LoVo cells.

d. Representative IHC staining images of HIF-1α, β-catenin, and TCF1 in CRC patients received preoperative fluorouracil analog-based chemotherapy. Scale bar = 100 μm.
