# Supplementary material for: ROS/PI3K/Akt and Wnt/β-catenin signalings activate HIF-1α-induced metabolic reprogramming to impart 5-fluorouracil resistance in colorectal cancer
Source: J Exp Clin Cancer Res. 2022 Jan 8;41:15. doi: 10.1186/s13046-021-02229-6 (PMC8742403; doi:10.1186/s13046-021-02229-6)
Supplement: Supplementary file 8 — Additional file 8: Table S1. Clinicopathological data. Clinicopathological data of CRC patients were gathered from the medical records and pathologic data. [file 13046_2021_2229_MOESM8_ESM.docx]

**Additional file 8. Table S1. Clinicopathological data.**

| **Patient Characteristics** | **Category** | **Frequency (number)** |
| --- | --- | --- |
| ***Cohort 1***  no chemotherapy group (without preoperative chemotherapy) | | |
| Age (years) | < 40 | 0.00% (0) |
|  | 40-49 | 26.67% (4) |
|  | 50-59 | 20.00% (3) |
|  | 60-69 | 33.33% (5) |
|  | 70-79 | 20.00% (3) |
|  | >80 | 0.00% (0) |
| Gender | Male | 53.33% (8) |
|  | Female | 46.67% (7) |
| T stage | T1 | 0.00% (0) |
|  | T2 | 6.67% (1) |
|  | T3 | 60.00% (9) |
|  | T4a | 33.33% (5) |
|  | T4b | 0.00% (0) |
| N stage | N0 | 0.00% (0) |
|  | N1a | 20.00% (3) |
|  | N1b | 13.33% (2) |
|  | N1c | 20.00% (3) |
|  | N2a | 26.67% (4) |
|  | N2b | 20.00% (3) |
| M stage | M0 | 93.33% (14) |
|  | M1 | 6.67% (1) |
| TNM stage | III | 93.33% (14) |
|  | IV | 6.67% (1) |
| ***Cohort 2***  response group (good response to preoperative fluorouracil analog–based chemotherapy) | | |
| Age (years) | < 40 | 0.00% (0) |
|  | 40-49 | 13.33% (2) |
|  | 50-59 | 33.33% (5) |
|  | 60-69 | 53.33% (8) |
|  | 70-79 | 0.00% (0) |
|  | >80 | 0.00% (0) |
| Gender | Male | 66.67% (10) |
|  | Female | 33.33% (5) |
| T stage | T1 | 6.67% (1) |
|  | T2 | 0.00% (0) |
|  | T3 | 60.00% (9) |
|  | T4a | 33.33% (5) |
|  | T4b | 0.00% (0) |
| N stage | N0 | 13.33% (2) |
|  | N1a | 13.33% (2) |
|  | N1b | 6.67% (1) |
|  | N1c | 13.33% (2) |
|  | N2a | 33.33% (5) |
|  | N2b | 20.00% (3) |
| M stage | M0 | 80.00% (12) |
|  | M1 | 20.00% (3) |
| TNM stage | III | 80.00% (12) |
|  | IV | 20.00% (3) |
| Primary chemotherapy regimens | 5-FU | 13.33% (2) |
|  | Capecitabine | 20.00% (3) |
|  | FOLFOX | 40.00% (6) |
|  | CAPEOX | 6.67% (1) |
|  | FOLFIRI | 13.33% (2) |
|  | FOLFOXIRI | 6.67% (1) |
|  | XELOX | 0.00% (0) |
| ***Cohort 3***  no response group (poor response to preoperative fluorouracil analog–based chemotherapy) | | |
| Age (years) | < 40 | 8.33% (1) |
|  | 40-49 | 25.00% (3) |
|  | 50-59 | 33.33% (4) |
|  | 60-69 | 16.67% (2) |
|  | 70-79 | 8.33% (1) |
|  | >80 | 8.33% (1) |
| Gender | Male | 58.33% (7) |
|  | Female | 41.67% (5) |
| T stage | T1 | 0.00% (0) |
|  | T2 | 0.00% (0) |
|  | T3 | 33.33% (4) |
|  | T4a | 66.67% (8) |
|  | T4b | 0.00% (0) |
| N stage | N0 | 0.00% (0) |
|  | N1a | 8.33% (1) |
|  | N1b | 50.00% (6) |
|  | N1c | 8.33% (1) |
|  | N2a | 8.33% (1) |
|  | N2b | 25.00% (3) |
| M stage | M0 | 75.00% (9) |
|  | M1 | 25.00% (3) |
| TNM stage | III | 75.00% (9) |
|  | IV | 25.00% (3) |
| Primary chemotherapy regimens | 5-FU | 8.33% (1) |
|  | Capecitabine | 8.33% (1) |
|  | FOLFOX | 41.67% (5) |
|  | CAPEOX | 0.00% (0) |
|  | FOLFIRI | 33.33% (4) |
|  | FOLFOXIRI | 0.00% (0) |
|  | XELOX | 8.33% (1) |
