# Supplementary material for: ROS/PI3K/Akt and Wnt/β-catenin signalings activate HIF-1α-induced metabolic reprogramming to impart 5-fluorouracil resistance in colorectal cancer
Source: J Exp Clin Cancer Res. 2022 Jan 8;41:15. doi: 10.1186/s13046-021-02229-6 (PMC8742403; doi:10.1186/s13046-021-02229-6)
Supplement: Supplementary file 9 — Additional file 9: Table S2. Reagent used in this study. [file 13046_2021_2229_MOESM9_ESM.docx]

**Additional file 9. Table S2. Reagent used in this study.**

| **REAGENT** | **SOURCE** | **IDENTIFIER** |
| --- | --- | --- |
| **Antibodies** | | |
| anti-CDK2 antibody | Proteintech | Cat# 10122-1-AP |
| anti-Cyclin D1 antibody | Proteintech | Cat# 26939-1-AP |
| anti-p21 antibody | Proteintech | Cat# 10355-1-AP |
| anti-Bcl-XL antibody | Proteintech | Cat# 26967-1-AP |
| anti-Cleaved caspase 3 antibody | Affinity | Cat# AF7022 |
| anti-Cleaved caspase 9 antibody | Affinity | Cat# AF5240 |
| anti-NDUFB8 antibody | Abcam | Cat# ab192878 |
| anti-SDHB antibody | Abcam | Cat# ab175225 |
| anti-UQCRC1 antibody | Proteintech | Cat# 21705-1-AP |
| anti-COX IV antibody | Abcam | Cat# ab202554 |
| anti-ATP5F1 antibody | Proteintech | Cat# 15999-1-AP |
| anti-GLUT1 antibody | Abcam | Cat# ab40084 |
| anti-GLUT2 antibody | Abcam | Cat# ab192599 |
| anti-GLUT3 antibody | Abcam | Cat# ab191071 |
| anti-GLUT4 antibody | Abcam | Cat# ab188317 |
| anti-MCT1 antibody | Abcam | Cat# ab85021 |
| anti-MCT2 antibody | Proteintech | Cat# 20355-1-AP |
| anti-MCT4 antibody | Santa | Cat# sc-376140 |
| anti-LDHA antibody | Abcam | Cat# ab52488 |
| anti-LDHA antibody | Proteintech | Cat# 66287-1-Ig |
| anti-HK2 antibody | Abcam | Cat# ab209847 |
| anti-GAPDH antibody | Proteintech | Cat# 60004-1-Ig |
| anti-ENO1 antibody | Abcam | Cat# ab227978 |
| anti-PKM2 antibody | CST | Cat# 4053 |
| anti-G6PD antibody | Abcam | Cat# ab133525 |
| anti-HIF-1α antibody | Abcam | Cat# ab179483 |
| anti-HIF-1α antibody | Proteintech | Cat# 66730-1-Ig |
| anti-Catalase antibody | Proteintech | Cat# 21260-1-AP |
| anti-GPx1 antibody | Abcam | Cat# ab108429 |
| anti-SOD1 antibody | Abcam | Cat# ab51254 |
| anti-SOD2 antibody | Abcam | Cat# ab68155 |
| anti-PI3K antibody | Affinity | Cat# AF6241 |
| anti-Phospho-PI3K antibody | Affinity | Cat# AF3241 |
| anti-AKT antibody | Abcam | Cat# ab179463 |
| anti-Phospho-AKT antibody | Abcam | Cat# ab38449 |
| anti-β-catenin antibody | Proteintech | Cat# 51067-2-AP |
| anti-Axin2 antibody | Abcam | Cat# ab109307 |
| anti-DVL1 antibody | Proteintech | Cat# 27384-1-AP |
| anti-TCF1 antibody | Proteintech | Cat# 22426-1-AP |
| anti-TCF4 antibody | Proteintech | Cat# 22337-1-AP |
| anti-Ki67 antibody | Proteintech | Cat# 27309-1-AP |
| anti-β-Actin antibody | Proteintech | Cat# 66009-1-Ig |
| anti-Lamin B1 antibody | Proteintech | Cat# 12987-1-AP |
| Rabbit IgG - Isotype Control | Abcam | Cat# ab172730 |
| **Chemicals** | | |
| 5-Fluorouracil | Abcam | Cat# ab142387 |
| 2-NBDG | MedChemExpress | Cat# HY-116215 |
| STF-31 | MedChemExpress | Cat# HY-18728 |
| AZD3965 | MedChemExpress | Cat# HY-12750 |
| Syrosingopine | MedChemExpress | Cat# HY-N4115 |
| D-Glucose | MedChemExpress | Cat# HY-B0389 |
| 3-Bromopyruvic acid | APExBIO | Cat# B7922 |
| 6-Aminonicotinamide | Selleck | Cat# S9783 |
| IDF-11774 | Selleck | Cat# S8771 |
| tert-Butylhydroperoxide | Sigma-Aldrich | Cat# 458139 |
| N-acetylcysteine | Selleck | Cat# S1623 |
| LY294002 | MedChemExpress | Cat# [HY-10108](https://www.medchemexpress.cn/LY294002.html) |
| MSAB | MedChemExpress | Cat# [HY-120697](https://www.medchemexpress.cn/msab.html) |
| Puromycin dihydrochloride | MedChemExpress | Cat# HY-B1743A |
| [Deuterium oxide](http://dict.youdao.com/w/deuterium%20oxide/#keyfrom=E2Ctranslation) | Sigma-Aldrich | Cat# [151882](https://www.sigmaaldrich.cn/CN/zh/product/aldrich/151882?context=product) |
| 3-(tetramethysilane) propionic acid-2,2,3,3-d4 | Sigma-Aldrich | Cat# 269913 |
| Succinic acid-^13^C_4_ | Sigma-Aldrich | Cat# 491985 |
| Glucose-^13^C_6_ | Cayman Chemical | Cat# 26707 |
| IRDye 800CW 2-DG Optical Probe | Li-Cor | Cat# 926-08946 |
| TRIzol reagent | TaKaRa | Cat# 279506 |
| **Critical Commercial Assays** | | |
| Cell counting kit-8 | MedChemExpress | Cat# HY-K0301 |
| Phalloidin-iFluor 488 reagent | Abcam | Cat# ab176753 |
| EdU incorporation assay kit | KeyGEN BioTECH | Cat# KGA331 |
| Cell cycle detection kit | KeyGEN BioTECH | Cat# KGA512 |
| Annexin V-PE/7-AAD apoptosis detection kit | KeyGEN BioTECH | Cat# KGA1016 |
| Mitochondrial staining kit | Abcam | Cat# ab112145 |
| Lactate release assay kit | KeyGEN BioTECH | Cat# KGT023 |
| Seahorse XF cell mito stress test kit | Agilent | Cat# 103010-100 |
| Seahorse XF glycolysis stress test kit | Agilent | Cat# 103020-100 |
| ATP assay kit | Beyotime | Cat# S0026 |
| \| Reactive oxygen species assay kit \| \| --- \| | Beyotime | Cat# S0033S |
| Lactate dehydrogenase assay kit (colorimetric) | Abcam | Cat# ab102526 |
| Catalase activity assay kit | Abcam | Cat# ab83464 |
| Glutathione peroxidase assay kit (colorimetric) | Abcam | Cat# ab102530 |
| Superoxide dismutase assay kit (colorimetric) | Abcam | Cat# ab65354 |
| Pierce co-immunoprecipitation kit | Thermo Fisher | Cat# 26149 |
| ReverTra Ace qPCR RT Kit | TOYOBO | Cat# FSQ-101 |
| SYBR Green Realtime PCR Master Mix | TOYOBO | Cat# QPK-201 |
| **Experimental Models** | | |
| Cell Line: HCT8 | KeyGEN BioTECH | Cat# KG028 |
| Cell Line: HCT15 | KeyGEN BioTECH | Cat# KG321 |
| Cell Line: HCT116 | KeyGEN BioTECH | Cat# KG167 |
| Cell Line: LoVo | KeyGEN BioTECH | Cat# KG027 |
| Cell Line: SW480 | KeyGEN BioTECH | Cat# KG019 |
| Cell Line: SW1116 | KeyGEN BioTECH | Cat# KG118 |
| Cell Line: HT29 | KeyGEN BioTECH | Cat# KG015 |
| Cell Line: Caco-2 | KeyGEN BioTECH | Cat# KG169 |
| Cell Line: DLD-1 | KeyGEN BioTECH | Cat# KG426 |
| Cell Line: T84 | KeyGEN BioTECH | Cat# KG448 |
| Cell Line: DiFi | Gift from Dr. Li Luo | N/A |
| Mouse: Athymic BALB/c nude mice (male) | SPF Biotechnology | N/A |
| Mouse: NOD/scid mice (male) | SPF Biotechnology | N/A |
