# Supplementary material for: ROS/PI3K/Akt and Wnt/β-catenin signalings activate HIF-1α-induced metabolic reprogramming to impart 5-fluorouracil resistance in colorectal cancer
Source: J Exp Clin Cancer Res. 2022 Jan 8;41:15. doi: 10.1186/s13046-021-02229-6 (PMC8742403; doi:10.1186/s13046-021-02229-6)
Supplement: Supplementary file 10 — Additional file 10: Table S3. Primers used in this study. [file 13046_2021_2229_MOESM10_ESM.docx]

**Additional file 10. Table S3. Primers used in this study.**

| **Primers used in RT-qPCR** | | |
| --- | --- | --- |
| **Gene** | **Forward Sequence** | **Reverse Sequence** |
| *NDUFB8* | CCACACCTGTTTCTTGGCATGTC | ATCACCGCCTCGTTCCAGGTAC |
| *SDHB* | GCAGTCCATAGAAGAGCGTGAG | TGTCTCCGTTCCACCAGTAGCT |
| *UQCRC1* | TGTCTCGTGCAGACTTGACCGA | GCGAGGTCTAACAGTTGCTGGT |
| *COX4I1* | TCGGTTTCACCGCGCTCGTTAT | TGTCCAGCATCCTCTTGGTCTG |
| *ATP5F1* | CTGTGCAGAACATGATGCGTCG | CTGTGCTTGAGCCTTCTTTGCC |
| *GLUT1* | TTGCAGGCTTCTCCAACTGGAC | CAGAACCAGGAGCACAGTGAAG |
| *GLUT2* | ATGTCAGTGGGACTTGTGCTGC | AACTCAGCCACCATGAACCAGG |
| *GLUT3* | TGCCTTTGGCACTCTCAACCAG | GCCATAGCTCTTCAGACCCAAG |
| *GLUT4* | CCATCCTGATGACTGTGGCTCT | GCCACGATGAACCAAGGAATGG |
| *MCT1* | TTGTTGGTGGCTGCTTGTCAGG | TCATGGTCAGAGCTGGATTCAAG |
| *MCT2* | TGCTGGCTGTTATGTACGCAGG | GCCAACACCATTCCAAGACAGC |
| *MCT4* | GCCATCTTTGCTGGTGGTTACC | TGGTCCAGAAAGGACAGCCATC |
| *G6PD* | CTGTTCCGTGAGGACCAGATCT | TGAAGGTGAGGATAACGCAGGC |
| *PGLS* | TGTGGCAACTGGAGAAGGCAAG | CTCGTCCAAGAACCAGCACAGT |
| *PGD* | GTTCCAAGACACCGATGGCAAAC | CACCGAGCAAAGACAGCTTCTC |
| *RPE* | AAGCCAATGGCTGTAGCAGGAG | CTGAGGTTCCTGGTTTGATGGC |
| *RPIA* | GTATGGCTTGACCCTCAGTGATC | CAGCCACAATCTTCTCCTGGGT |
| *TKT* | CCAAGTGATGGCGTTGCTACAG | TTGTCCGACCTGGAAGTCCTCA |
| *TKTL1* | CCTGGAGGATATAGCCATGTTCC | GTGGTCCGAATGAAGCACATCC |
| *TKTL2* | AATCGCCTGTGGAAGACTCACC | GCCCAGTTTAGCCAGAGCCAAA |
| *TALDO1* | TGCCTGTGCTCTCAGCCAAGG | TTCTCCACAGCCATCTGGTCCT |
| *HK2* | GAGTTTGACCTGGATGTGGTTGC | CCTCCATGTAGCAGGCATTGCT |
| *GPI* | CTGGTAGACGGCAAGGATGTGA | TCCGTGATGGTCTTGCCTGTGT |
| *PFKFB2* | TACCGACCTCTTGACCCAGACA | TAAATGGTGCGAGGCTGGACGT |
| *PGK1* | CCGCTTTCATGTGGAGGAAGAAG | CTCTGTGAGCAGTGCCAAAAGC |
| *ENO1* | AGTCAACCAGATTGGCTCCGTG | CACAACCAGGTCAGCGATGAAG |
| *PDK1* | CATGTCACGCTGGGTAATGAGG | CTCAACACGAGGTCTTGGTGCA |
| *LDHA* | GGATCTCCAACATGGCAGCCTT | AGACGGCTTTCTCCCTCTTGCT |
| *CAT* | GTGCGGAGATTCAACACTGCCA | CGGCAATGTTCTCACACAGACG |
| *GPX1* | GTGCTCGGCTTCCCGTGCAAC | CTCGAAGAGCATGAAGTTGGGC |
| *SOD1* | CTCACTCTCAGGAGACCATTGC | CCACAAGCCAAACGACTTCCAG |
| *SOD2* | CTGGACAAACCTCAGCCCTAAC | AACCTGAGCCTTGGACACCAAC |
| *HIF1A* | TATGAGCCAGAAGAACTTTTAGGC | CACCTCTTTTGGCAAGCATCCTG |
| *ACTB* | CACCATTGGCAATGAGCGGTTC | AGGTCTTTGCGGATGTCCACGT |
| **shRNA coding sequence** | | |
| **Gene** | **Target sequence** | |
| sh*HIF1A* | aaTGTGAGTTCGCATCTTGAT  (Vector: hU6-MCS-Ubiquitin-EGFP-IRES-puromycin, Jikai Gene, China) | |
| **siRNA sequences targeting genes** | | |
| **Gene** | **Sense** | **Anti-sense** |
| si*HIF1A* | UACUCAGAGCUUUGGAUCAAGUUAA | UUAACUUGAUCCAAAGCUCUGAGUA |
| *s*i*CTNNB1* | GCUCAUCAUACUGGCUAGUTT | ACUAGCCAGUAUGAUGAGCTT |
